# Alternative splicing level related to intron size and organism complexity

**DOI:** 10.1186/s12864-021-08172-2

**Published:** 2021-11-25

**Authors:** Pengcheng Yang, Depin Wang, Le Kang

**Affiliations:** 1 Beijing Institutes of Life Science, Chinese Academy of Sciences, Beijing, China; 2 Sino-Danish College, University of Chinese Academy of Sciences, Beijing, China

**Keywords:** RNA-Seq, Protein disorder, Intron density, Splicing factor, Conservation, Gene family

## Abstract

**Background:**

Alternative splicing is the process of selecting different combinations of splice sites to produce variably spliced mRNAs. However, the relationships between alternative splicing prevalence and level (ASP/L) and variations of intron size and organism complexity (OC) remain vague. Here, we developed a robust protocol to analyze the relationships between ASP/L and variations of intron size and OC. Approximately 8 Tb raw RNA-Seq data from 37 eumetazoan species were divided into three sets of species based on variations in intron size and OC.

**Results:**

We found a strong positive correlation between ASP/L and OC, but no correlation between ASP/L and intron size across species. Surprisingly, ASP/L displayed a positive correlation with mean intron size of genes within individual genomes. Moreover, our results revealed that four ASP/L-related pathways contributed to the differences in ASP/L that were associated with OC. In particular, the spliceosome pathway displayed distinct genomic features, such as the highest gene expression level, conservation level, and fraction of disordered regions. Interestingly, lower or no obvious correlations were observed among these genomic features.

**Conclusions:**

The positive correlation between ASP/L and OC ubiquitously exists in eukaryotes, and this correlation is not affected by the mean intron size of these species. ASP/L-related splicing factors may play an important role in the evolution of OC.

**Supplementary Information:**

The online version contains supplementary material available at 10.1186/s12864-021-08172-2.

## Background

Alternative splicing plays important roles in the functional diversity of proteins in higher eukaryotic genomes. Alternative splicing allows one gene to generate functionally distinct isoforms, which is an important driving force for increasing organism complexity (OC) [1]. The alternative splicing prevalence (ASP) is influenced by OC and intron size [2, 3]. Approximately 95% of human intron-containing genes undergo alternative splicing [4], but this ratio is 60.7% in the fruit fly and only 2.9% in green alga [5, 6]. Comparative analyses revealed that species with higher OC have higher ASP [3, 7–10]. In addition, several studies found that both ASP and alternative splicing level (ASL) are positively correlated with intron size among genes within individual genomes [7, 10–12]. Generally, intron size is positively correlated with genome size in animals [13]. However, genome size, as measured by the C-value, does not strongly correspond to OC, which causes the C-value paradox [14]. Therefore, whether the relationship between ASP and ASL (ASP/L) and OC is confounded by intron size remains unclear.

OC is often measured as the cell type number (CTN) per species [15, 16], and has been widely used in studies to investigate the relationship between OC and genomic features [3]. OC is clearly correlated with several genomic features, including ASP/L [3], the fraction of disordered residues in proteins [8, 17], degree of protein−protein interactions [8], total number of transcription factors [18] and average number of their isoforms [19], functional diversity [20], and proteome size [8, 17]. Moreover, it is known that the splicing factors interact with other RNA-binding proteins to regulate alternative splicing through binding to the cis-regulatory elements on the gene body [21]. However, whether the splicing factors contribute to OC and is linked to numbers of genes and numbers of disordered residues remains unknown.

In the past two decades, some studies have compared ASP/L across species. Different tissues were found to display substantially different splicing levels [22], and genes with higher expression levels tend to undergo more splicing [23]. In addition, isoform/splicing events can be measured at three levels, the isoform, exon, and intron levels, by level-specific software packages, and each of these approaches has distinct merits and drawbacks [24]. There have been extensive comparisons of ASP/L across species based on expressed sequence tag data [2, 3, 7, 8, 25–27]. With the reductions in sequencing costs in recent years, several studies have been able to directly compare ASP/L across multiple species in a single experiment by performing RNA-Seq on the same tissues of closely related species, applying the same analysis methods, and basing the calculations of ASP/L on equal quantities of orthologous exon or intron data [9, 28–30]. However, because none of the orthologs showed complete conservation of splicing structures among distantly related species, direct comparisons between distantly related organisms such as deuterostomes and protostomes are still impossible [31]. In addition, although subsampling of 20× sequencing depth data to estimate species-level ASP is performed across remote species, 20× sequencing depth is far from saturation [23].

In this study, we used both ASP and ASL measurements. ASP denotes the proportion of multi-exon genes with more than one isoform, whereas ASL denotes the average number of isoforms per multi-exon gene. To remove the effect of intron size on the relationships between ASP/L and OC, we designed three sets of species. The first set of species (FirstSpeciesSet) covered the species with larger introns (2−42 Kb) and extremely varied OC (CTN, 50−216). The second set of species (SecondSpeciesSet) covered the species with smaller intron sizes (0.7−3 Kb) and extremely varied OC (CTN, 22−119). The third set of species (ThirdSpeciesSet) included species with a fixed OC (CTN, 59) but greatly varying intron sizes (0.3−14 Kb), and was comprised of dipteran insect species. The three sets of species included ~8 Tb of raw RNA-Seq data from 37 eumetazoan species, and were analyzed by three isoform/splicing event identification methods, expression level-based binning, both ASP and ASL measurements, and a corrective calculation method. We found common regulation of ASP/L relative to OC and intron size.

## Results

### Experimental design

**Fig. 1:**
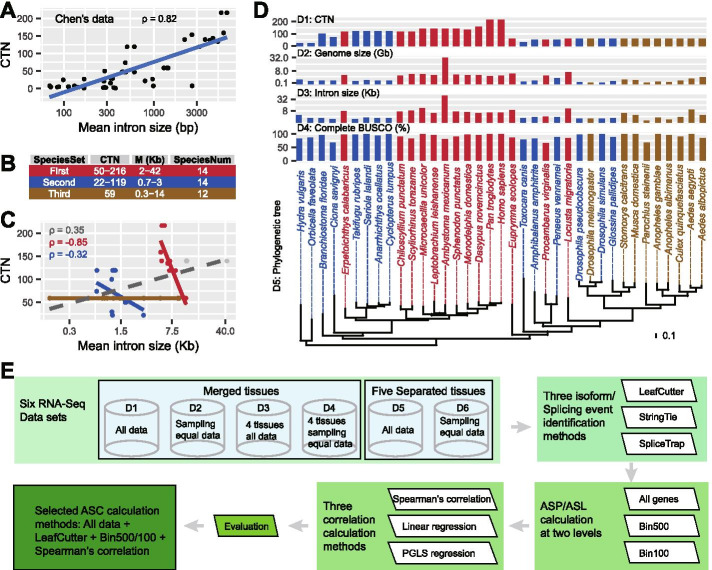
Experimental design. (**A**) The organism complexity (OC) of 50 species exhibited a positive correlation with their mean intron size (Spearman’s *ρ* = 0.82, *P* = 7.6e-13) in [3]. (**B**) Statistics of the three sets of species constructed in the present study. CTN, cell type number; M, mean intron size (Kb). (**C**) The distribution of mean intron size versus the CTN of the 37 species used in the present study. The mean intron size was positively correlated with CTN across all 37 species (gray line; Spearman’s *ρ* = 0.35, *P* = 0.02), and negatively correlated with CTN in the FirstSpeciesSet [all species (red and gray data points), Spearman’s *ρ* = −0.23, *P* = 0.24; without Ame, Mun, and Pvi (three gray data points), Spearman’s *ρ* = −0.85, *P* = 8e-04] and the SecondSpeciesSet (blue points; Spearman’s *ρ* = −0.32, *P* = 0.26). (**D**) Information regarding the 37 selected species, including their CTN (D1), genome size (D2), intron size (D3), percentage of complete BUSCO (D4), and phylogenetic tree (D5). The three colors used for text and bars represent the three sets of species and are the same in (**B**) and (**C**). Three species, Dps, Dsim, and Gpa, were shared among the Second- and ThirdSpeciesSet. (E) Workflow of the analysis used to explore the correlation between alternative splicing prevalence/level (ASP/L) and OC using the FirstSpeciesSet. This procedure included six datasets, three splicing event/isoform identification methods, two ASP and ASL calculation strategies, and three correlation-level calculation methods. Following our evaluation, we selected the following strategies for ASP/L calculation: LeafCutter was used to analyze all RNA-Seq data, calculate ASP/L using Bin500/100 genes, and calculate the correlation level using Spearman’s correlation

We first reanalyzed the species used in Chen’s study [3], in which 112 eukaryotes were used and they found that the ASP/L is positively correlated with OC, and we found that the mean intron size is positively correlated with OC (Fig. 1 A; Spearman’s *ρ* = 0.82, *P* = 7.6e-13). We thus argue that the positive correlation between ASP/L and OC may be confounded by intron size. To assess whether ASP/L is determined by OC or intron size, we compiled three sets of species (Fig. 1B, C, and D) from 37 species. These species were selected because first of all they have high-quality genome assemblies (Scaffold N50 > 200 Kb; Additional file 1: Table S1) and gene annotations (complete BUSCO > 80%; Fig. 1D), and adequate RNA-Seq data (unique mapped base > 16 Gb; Additional file 2: Table S2). To select as many species as possible, two species (Pvi and Pst) that don’t satisfy these criteria were included, and the alternative results without these species were specifically denoted. Secondly, these species satisfy our requirements for the relationship between intron size and OC (see later). We found the mean intron size was also positively correlated with OC across these 37 species (Fig. 1 C; Spearman’s *ρ* = 0.35, *P* = 0.02). We collected approximately 8 Tb of RNA-Seq data that were mapped to their corresponding genomes (Additional file 1: Table S1 and Additional file 2: Table S2). Evaluation with BUSCO indicated that these three sets of species had high-quality gene annotations (Fig. 1D).

The first set of species (FirstSpeciesSet) was comprised of 14 bilaterian species, which included 11 deuterostome species (such as *Homo sapiens*) and three protostome species. This species set has extremely varied OC (CTN, 50−216), larger introns (> 2 Kb), and the intron size varied 21-fold (2–42 Kb) (Fig. 1 C; Additional file 1: Table S1.1). Especially, all non-human species had lower OC but larger genome sizes than the human genome. As expected, these 14 species displayed a negative correlation between mean intron size and CTN (all species, Spearman’s ρ = −0.23, *P* = 0.24; without Ame, Mun, and Pvi, ρ = −0.85, *P =* 8e-04; Fig. 1 C).

Because the splicing mechanism differs among exons that are flanked by differently sized introns [7], we constructed the second set of species (SecondSpeciesSet) with a mean intron size ranging from 700 bp to 3 kb and an extremely varied OC (CTN, 22−119). We found a negative correlation between OC and the mean intron size (Spearman’s ρ = −0.32, *P =* 0.26; Fig. 1B and C; Additional file 1: Table S1.2).

To remove the impact of OC on the investigations of the relationship between ASP/L and intron size, for the ThirdSpeciesSet, we selected 12 dipteran species with the same OC (CTN, 59) but greatly varying intron size (0.3−14 Kb; Fig. 1B and C; Additional file 1: Table S1.3). These 12 species displayed 18-fold genome size variation (2247 Mb/125 Mb) and 41-fold intron size variation (347–14,117 bp).

To minimize the impact of several potential sample and technological biases on alternative splicing event identification, such as sequencing depth, tissue type, software used, and gene expression level, we designed a strategy to combine the datasets and analysis methods and applied it to the FirstSpeciesSet (see Materials and Methods; Fig. 1E; Additional file 3: Figure S1 and Additional files 4, 5, 6 and 7: Table S3-6). Generally, we constructed six RNA-Seq datasets according to the sequencing depth and tissue type, and named D1 to D6, respectively. We employed three representative tools to identify isoform/splicing events: LeafCutter, StringTie, and SpliceTrap. We used two bin sizes, 100 and 500, to measure the influence of sequencing depth to the ASP/L. We also utilized three methods to calculate the correlation between ASP/L and OC: Spearman, linear regression, and phylogenetic generalized least squares (PGLS) regression.

### ASP/L is positively correlated with OC

**Fig. 2:**
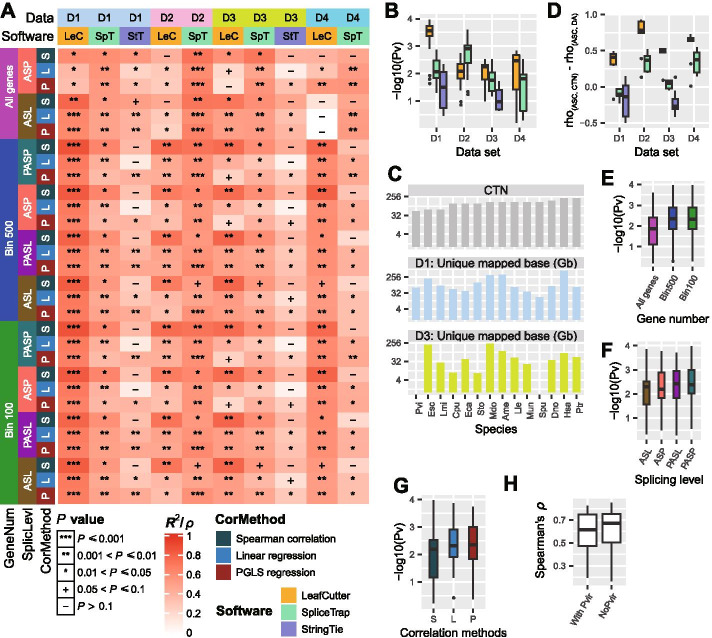
ASP/L is positively correlated with organism complexity in the FirstSpeciesSet. (**A**) The 10 columns are combinations of four datasets (D1–D4) and three tools (LeafCutter, SpliceTrap, and StringTie). StringTie was not run for the D2 and D4 datasets because it failed to construct full-length transcripts as a result of the uneven distribution of the sampled reads. The 30 rows are combinations of the three numbers of genes used for ASP/L calculation (all genes, Bin 500, and Bin 100), two statistics for alternative splicing level [alternative splicing prevalence (ASP) and alternative splicing level (ASL); for the binned genes, we also calculated the predicted ASP (PASP) and ASL (PASL)], and three correlation calculation methods [Spearman’s correlation, linear regression, and phylogenetic general least squares (PGLS) regression]. The colors of the cells represent the Spearman’s correlation or adjusted R-squared value of the linear regression and PGLS regression. *P ≤ 0.05, **P ≤ 0.01, ***P ≤ 0.001, +*P* ≤ 0.1, and −*P* > 0.1. (**B**) Distribution of the *P* values in (**A**) across the four datasets and three tools. (**C**) Comparisons of cell type number (CTN) and data quantity in the D1 and D3 datasets of the 14 species. (**D**) Distribution of the correlation differences between the Spearman’s ρ between ASP/L and CTN, and between ASP/L and data quantity. (**E**) Distributions of *P* values calculated using the three gene selections (all, Bin 500, and Bin 100). (**F**) Distribution of *P* values calculated using the four measurements of ASP/L. (**G**) Distribution of *P* values calculated using the three correlation methods. (**H**) Boxplots of Spearman’s ρ between ASP/L and CTN using either all 14 species or 13 species without Pvi. The mean ρ values with and without Pvi were 0.61 and 0.59, respectively

In general, we found a significantly positive correlation between ASP/L and OC in the FirstSpeciesSet (Fig. 2 A; Additional file 6: Table S5.1). Combining all datasets and analyses methods, 80% of the correlations had *P* values < 0.05. We used these *P* values of the correlation to evaluate the impact of different datasets and methods on the calculation of ASP/L. We did not find any dataset that outperformed all other datasets across all combinations of methods. However, the datasets that contained more data and included a larger number of different tissues showed higher significance levels (Fig. 2B; e.g., D1 vs. D3, student’s *t*-test, *P* < 0.01 for all three tools; D1_LeafCutter vs. LeafCutter for all three of the other datasets). We also observed that D2_SpliceTrap exhibited higher significance than D1_SpliceTrap (student’s *t*-test, *P* < 0.001), but there was no difference between D3 and D4 (Fig. 2B). We speculated that the difference between D2_SpliceTrap and D1_SpliceTrap resulted from the different quantities of data included from the 14 species that comprised each dataset. However, no correlations were found between CTN and data quantity in D1 and D3 (Fig. 2 C; Spearman’s ρ = 0.09, *P* = 0.75 in D1; Spearman’s ρ = 0.21, *P* = 0.52 in D3). Therefore, we inferred that the correlation between ASP/L and CTN would be greater than the correlations between CTN and data quantity in D1 and D3. Surprisingly, we found that most of the ASP/L values calculated using StringTie and SpliceTrap had a higher ρ value relative to the data quantity than the CTN (Fig. 2D). However, the ASP/L values calculated using LeafCutter for datasets D2 and D4 were not influenced by variations in data quantity. To eliminate the effect of data quantity, we excluded the results of StringTie and SpliceTrap for D1 and D3 from later analyses.

The *P* values of the correlations calculated using genes selected based on the binning method were more significant than those calculated using all of the genes (Fig. 2E; student’s *t*-test, *P* < 0.015 for both binning methods), which indicated that the ASP/L values estimated using highly expressed genes were more accurate than those estimated using all genes. We also observed that the *P* values of correlations calculated using the predicted ASP or ASL (PASP or PASL) based on the binning method were slightly more significant than those calculated using the actual ASP or ASL [Fig. 2 F; mean −log_10_ *P* values from the PASP and actual ASP, 2.5 and 2.3, respectively; mean −log_10_ *P* values from PASL and actual ASL, 2.4 and 2.2, respectively], which indicated that the model used for fitting the data was suitable for the ASP/L calculations. The positive correlation between ASP/L and OC was still present after correcting for phylogenetic distance and displayed a slightly higher level of significance than Spearman’s correlation (Fig. 2G). Based on the above evaluation, we selected LeafCutter to analyze all datasets from the SecondSpeciesSet and ThirdSpeciesSet, the determination of ASP/L using bins of 100 or 500 genes, and calculation of the correlation using Spearman’s correlation.

**Fig. 3:**
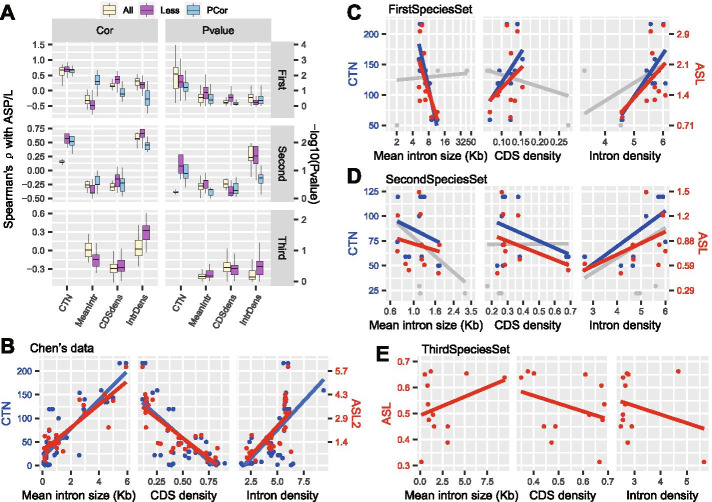
ASP/L does not correlate with intron size among genomes. (**A**) Distribution of Spearman’s ρ and *P* values of the correlations between ASP/L and CTN plus the three intron size-related statistics for the three sets of species. The correlation was calculated using all species (blue) or fewer species (pink). For the ThirdSpeciesSet, the fewer species calculation excluded Pst. The blue boxes represent the partial correlation produced by removing the effect of CTN or mean intron size. (**B**) The scatter plots depict the correlation between three intron size-related statistics, mean intron size (left), coding sequence (CDS) density (middle), and intron density (right), and two species-level complexity proxies, organism complexity (CTN; the blue points and the y-axis on the left) and alternative splicing level (ASL2; the red points and the y-axis on the right) from Chen’s data [3]. (**C**) Same analysis as in (**B**) but only using species from the FirstSpeciesSet. The gray data points represent the three species that were excluded because of the outlier relationship between mean intron size and CTN. The gray line represents the linear regression line that was fitted using all of the species. The three excluded species were Ame, Pvi, and Mun. ASL was calculated using the LeafCutter tool and a Bin500 gene number. (D) Same analysis as in (**B**) but only using species from the SecondSpeciesSet. The three excluded species were Ofa, Hvu, and Tca. The Spearman’s ρ in (**B**–**D**) was illustrated in Additional file 11: Figure S4. (**E**) Same analysis as in (**B**) but using only species from the ThirdSpeciesSet with fixed organism complexity

We also recalculated the Spearman’s ρ between ASP/L and CTN after removing species Pvi, which was the only species with a percentage of complete BUSCO < 80% (65.9%, Fig. 1D4). Spearman’s ρ displayed a similar distribution when including or excluding Pvi (Fig. 2 H). After removing the effect of intron size, Spearman’s ρ remained unchanged (Fig. 3 A). To produce a sharper contrast between OC and mean intron size, we removed three outlier species, Ame, Mun, and Pvi. The residual 11 species produced a significant negative correlation between OC and mean intron size (Spearman’s ρ = −0.85, *P =* 8e-04; Fig. 1 C). InterestinglyTherefore, the correlation coefficient between ASP/L and OC calculated using the 11 species was a little higher than that calculated using 14 species (0.61 vs. 0.57; Fig. 3 A). These results indicated that our data and the methods used to calculate the correlations with ASP/L were reliable and that the positive correlation between ASP/L and OC was robust.

We found that the positive correlation between ASP/L and OC was also present at the tissue-level, especially for brain tissue (Additional file 8: Figure S2). Interestingly, we found that the brain had the highest ASP/L, followed by testis tissue (Additional file 9: Table S7).

When we analyzed the SecondSpeciesSet (Additional file 1: Table S1.2, Additional file 2: Table S2.2, and Additional file 6: Table S5.2), we did not find a significant correlation between ASP/L and OC. Surprisingly, we found three species with lower OC but higher ASP/L values (Ofa, Hvu, and Tca; Additional file 10: Figure S3). For example, Hvu and Tca had 22 and 29 cell types, but their ASPs (0.79 and 0.80, respectively) were higher than the mean ASP (0.74) of seven species for which the CTN was 119; this is probably due to species-specific genetic features, which are discussed later. After removing these three outlier species, ASP/L showed a positive correlation with OC (Fig. 3 A). Upon combining these three species sets, we observed a positive correlation between OC and ASP/L (Additional file 10: Figure S3A; Spearman’s ρ = 0.63, *P* = 1.27e-05).

### ASP/L does not correlate with intron size across genomes

To investigate the relationship between ASP/L and mean intron size across the genomes in the two sets of species, FirstSpeciesSet and SecondSpeciesSet, we simultaneously tested another two intron size-related statistics, the coding sequence (CDS) density (mean CDS length/gene length) and intron density (introns per kb exon). Surprisingly, we found that the correlation between these three intron size-related statistics and ASP/L co-varied with OC (Fig. 3B−D; Additional file 11: Figure S4) in both our sets of species and those of a previous study [3]. The correlation levels calculated using the combinations of the above-mentioned six datasets and various methods were also consistent with this result (Fig. 3 A). The two sets of species showed negative correlations between mean intron size and OC. Interestingly, we also observed a negative correlation between mean intron size and ASP/L in both sets of species, a positive correlation between CDS density and ASP/L in the FirstSpeciesSet, and a negative correlation between CDS density and ASP/L in the SecondSpeciesSet. The positive correlations between intron density and both OC and ASP/L were not influenced by the datasets (Fig. 3 A−D). In both the First- and SecondSpeciesSet, the positive correlation between ASP/L and OC had the highest ρ and significance among the comparisons of ASP/L with CTN and the three intron size-related statistics. These correlations remained robust after removing the outlier species and the impact of mean intron size (Fig. 3 A). Conversely, the correlations between ASP/L and the three intron size-related statistics displayed lower ρ values that were around zero and *P* values that were mostly > 0.1. These results suggest that the relationships between ASP/L and the intron size-related statistics were confounded by OC.

To further remove the impact of OC on the calculation of relationships between ASP/L and intron size, we calculated ASP/L by using LeafCutter (Additional file 2: Table S2.3) based on the ThirdSpeciesSet from 12 dipteran species. Interestingly, although the intron size varied markedly, ASP/L did not correlate with any of the three intron size-related statistics (Fig. 3 A and E; Additional file 6: Table S5-3). After removing Pst, for which the complete BUSCO was 67.4%, the Spearman’s ρ between OC and the intron size-related statistics varied somewhat but was still not significant (*P* > 0.1; Fig. 3 A). After combining these three species sets, we observed a weak positive partial correlation between mean intron size and ASL with controlling OC (Additional file 10: Figure S3B; Spearman’s ρ = 0.33, *P* = 0.04). In contrast, the partial correlation between ASL and OC was higher and more significant (Spearman’s ρ = 0.55, *P* = 2.8e-4).

As highly expressed genes tend to have shorter introns [32] and the ASP/L calculated using the binning method employed highly expressed genes, we recalculated the correlation of ASP/L with the three intron size-related statistics using the same genes as those used for the ASP/L calculation. We found that the Spearman’s ρ values calculated using the highly expressed genes were distributed similarly to those using all genes (Additional file 12: Figure S5). Taken together, these results suggest that there is no correlation between ASP/L and intron size-related statistics across genomes.

### ASP/L is significantly correlated with intron size within individual genomes

**Fig. 4:**
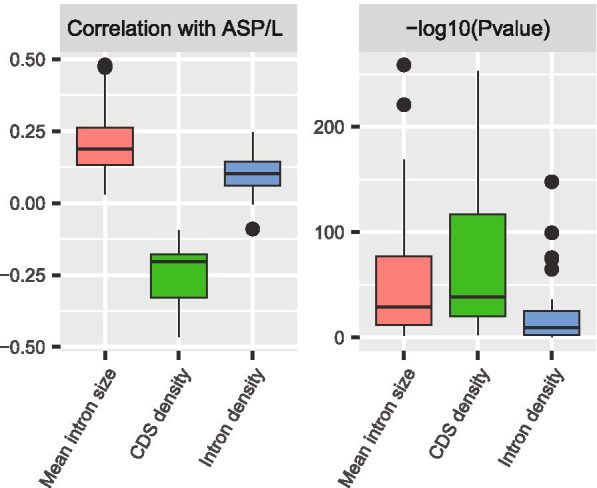
ASP/L is significantly correlated with the three intron size-related statistics within individual genomes. After combining the three sets of species and removing redundancy, a total of 37 species were used for this analysis

Using the highly expressed genes, we investigated the correlations between ASP/L and the three intron size-related statistics among genes within individual genomes. Combining the three sets of species and removing redundancy, 37 species were included in this analysis. We found that ASP/L was positively correlated with mean intron size and intron density, and was negatively correlated with CDS density (Fig. 4).

### Genomic features of ASP/L-related pathways are positively correlated with OC

Why do the significant correlations between ASP/L and intron size-related statistics exist within individual genomes, but not exist across genomes with different OC? We speculated that this phenomenon may be attributable to splicing factors, because they regulate the splicing process through binding to the cis-regulatory elements on the gene body. To verify this, we generated a dataset comprising genes from four ASP/L-related Kyoto Encyclopedia of Genes and Genomes (KEGG) pathways, namely Spliceosome (ko03040), mRNA Surveillance (ko03015), RNA Degradation (ko03018), and RNA Transport (ko03013). Six genomic features of these genes were analyzed and their relationships to OC were investigated. These six genomic features included gene expression level, number of orthologous genes, protein evolutionary distance, ASP/L, fraction of disordered residues, and fraction of disordered binding residues.

**Fig. 5:**
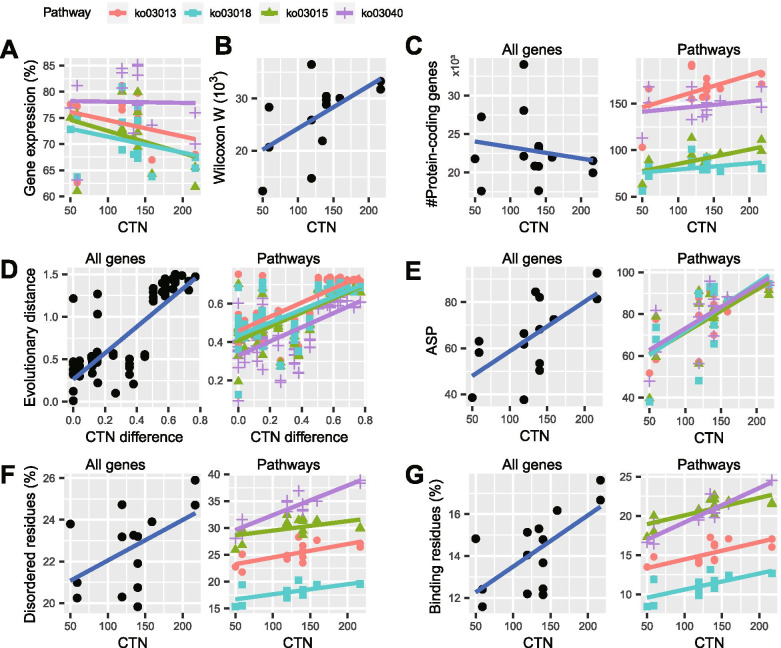
Genomic features of four ASP/L-related pathways are positively correlated with organism complexity in the FirstSpeciesSet. The four pathways investigated were RNA transport (ko03013), mRNA surveillance pathway (ko03015), RNA degradation (ko03018), and spliceosome (ko03040). The gene expression levels were calculated using D1 LeafCutter data (**A**), and for each species, the Wilcoxon W-statistics was calculated between ko03040 and the combination of the other three pathways (**B**). (**C**) Scatter plots of the genome-wide number of protein-coding genes (left) and that of the four pathways (right) vs. the cell type number (CTN). (**D**) Scatter plots of the evolutionary distance and organism complexity differences across the 14 species based on the phylogenetic tree shown in Fig. 1 A (left) and the protein alignment of the KEGG Orthologs from the four pathways (right). The CTN difference was calculated as diff(a,b)/max(a,b) and ranged between 0 and 1. (**E**) Scatter plots of alternative splicing prevalence and CTN using the D1 dataset and LeafCutter tool for all genes (left) or the genes from the four pathways (right). (**F**) Scatter plots of the fraction of disordered residues vs. CTN for all genes (left) and genes along the four pathways (right). (**G**) Scatter plots of the fraction of disordered binding residues vs. CTN for all genes (left) and the genes along the four pathways (right)

Interestingly, in the FirstSpeciesSet, we found that the expression levels of genes along the spliceosome pathway were higher than those of the genes of the other three pathways (Fig. 5 A). Furthermore, the expression levels of genes along the spliceosome pathway did not change with increasing OC, whereas the genes along the other three pathways showed decreasing expression with increasing OC. Thus, the gene expression differences between the spliceosome pathway and the combination of the other three pathways increased significantly with increasing OC (Fig. 5B; Spearman’s ρ between Wilcoxon W-statistic and CTN = 0.7, *P* = 0.004; see the figure legend for the detail method). The results were supported by expression data from different datasets and tools (Additional file 13: Figure S6).

We found that more complex species tend to have more orthologous ASP/L-related genes with their total gene number as the background (Fig. 5 C; Chi-square test: ko03013, *P* = 2.5e-08; ko03040, *P* = 9.3e-7; ko03015, *P* = 7.2e-04; ko03018, *P* = 2.7e-03). We found that the evolutionary distance based on the species tree displayed a strong positive correlation with OC (Fig. 5D left; Spearman’s ρ = 0.78, *P* = 4.7e-20). The evolutionary distances calculated using the genes specific to the four pathways also displayed similar correlation levels (Fig. 5D right; for the four pathways, 0.63 < Spearman’s ρ < 0.65, 2.3e-12 < *P* < 2.7e-11). Interestingly, the spliceosome pathway showed the shortest evolutionary distance (Fig. 5D right), which indicated that this pathway is under high evolutionary pressure. Using the D1 dataset and applying the LeafCutter tool to all genes, we found a positive correlation between ASP and OC (Fig. 5E left; Spearman’s ρ = 0.6, *P* = 0.02). Using the same dataset and method, analysis of the four pathways displayed similar distributions, with the spliceosome pathway having the highest correlation coefficient (Fig. 5E right; Spearman’s ρ for ko03040, ko03013, ko03018, and ko03015 were 0.71, 0.71, 0.66, and 0.56, the *P* values were 4.3e-03, 4.7e-03, 9.5e-03, and 3.7e-02, respectively).

We observed a positive correlation between the fraction of disordered residues and OC using all of the protein-coding genes (Fig. 5 F left; Spearman’s ρ = 0.36, *P* = 0.2). The four pathways displayed similar patterns, with the spliceosome pathway having the highest correlation coefficient (Fig. 5 F right; Spearman’s ρ for ko03040, ko03013, ko03018, and ko03015 were 0.72, 0.64, 0.60, and 0.28, the *P* value were 3.5e-03, 1.3e-02, 2.4e-02, and 0.33, respectively). For the fraction of disordered binding residues, we observed a positive correlation between this parameter and OC when using all of the protein-coding genes (Fig. 5G left; Spearman’s ρ = 0.53, *P* = 0.05). The four pathways showed similar patterns, with the spliceosome pathway having the highest correlation coefficient (Fig. 5G right; Spearman’s ρ for ko03040, ko03013, ko03018, and ko03015 were 0.83, 0.68, 0.66, and 0.73, the *P* values were 2.6e-04, 6.9e-03, 1.1e-02, and 2.9e-03, respectively).

The SecondSpeciesSet displayed similar relationships between OC and these genomic features (Additional file 14: Figure S7). These results suggest that the four ASP/L-related pathways, especially the spliceosome pathway, have significant correlations with OC.

### Genomic features of ASP/L-related pathways independently regulate OC

**Fig. 6:**
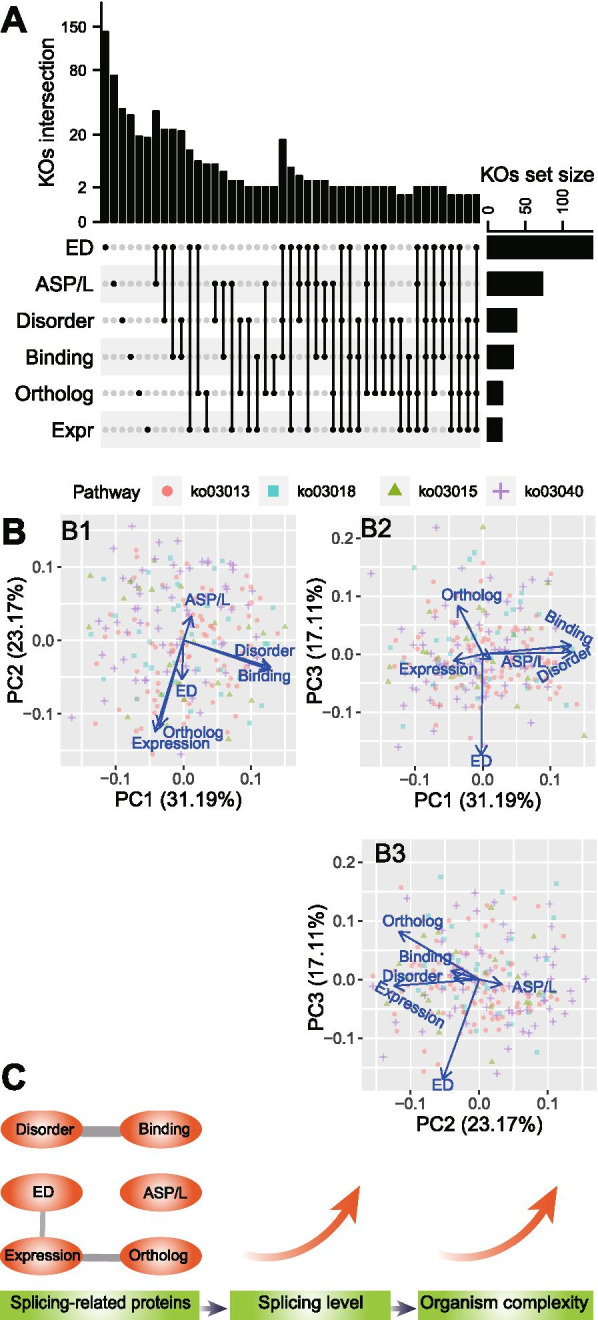
Six genomic features of ASP/L-related genes independently regulate organism complexity. (**A**) Intersection of significant KEGG Orthologs (KOs) among six genomic features. Spearman’s correlation and phylogenetic general least squares (PGLS) regression were calculated between these genomic features and organism complexity for each KO in the FirstSpeciesSet of 14 species. Significant KOs were defined as having Spearman’s ρ > 0.4 and PGLS *P* < 0.05. (**B**) Principal component analysis of the six genomic features based on the Spearman’s ρ for 219 KOs. The data points represent KOs and the shapes represent the four KEGG pathways. (**C**) Summary of the correlations among ASP/L, the six genomic features of ASP/L-related proteins, and organism complexity

To investigate whether some specific gene families (KEGG Orthology: KO) from the four ASP/L-related pathways displayed a higher correlation with OC across most of these six genomic features, we set the PGLS *P* < 0.05 and Spearman’s ρ > 0.4 as the cut-offs for filtering the results. In the FirstSpeciesSet, we annotated 316 KOs from the ASP/L-related pathways; approximately 208 of them had significant correlations with OC for at least one genomic feature, and 13 KOs appeared to have significant correlations with OC across at least three features (Fig. 6 A; Additional file 15: Table S8.1−7). Surprisingly, we did not find any KOs that were significant across all six features, and only one KO was significant across five features. The genomic features are probably independent of each other, and the principal component analysis using the Spearman’s ρ between each genomic feature and OC for the 219 KOs supported this hypothesis (Fig. 6B). We found that these six features were separated well using the first three principal components, except for the group containing the fractions of disordered binding residues and disordered residues. The SecondSpeciesSet displayed a similar pattern (Additional file 16: Figure S8).

## Discussion

Our results indicated that the positive correlation between ASP/L and OC is a general feature of eukaryotes. We found that the positive correlation was independent of intron size. Therefore, our present study largely extended the relevant conclusion as listed below [3].

Our study indicated that ASP/L is not always correlated with intron size-related statistics among species. We found no correlation between ASP/L and intron size across species based on evidence from three sets of species with varied or fixed OC. This result is different from the results of a previous study derived from a set of species with a positive correlation between intron size and OC [2]. Intuitively, it is reasonable that Lmi and Ame should have a lower ASP/L than humans even though their introns are longer (Additional file 6: Table S5), because their organism complexities are lower than humans.

Intriguingly, however, several species had lower OC accompanied by a higher ASP/L than expected. For Hvu, there are currently no species from the same order or class that satisfied our requirements regarding the quality of genome assembly and gene annotation or the quantity of RNA-Seq data available. It is thus unclear whether a high ASP level is ubiquitous within the Hydrozoa class or specific to Hvu. Tca is from the same order, Rhabditida, as *Caenorhabditis elegans*, for which ASP is only 0.25 [33]. Therefore, the higher level of ASP in Tca is species-specific. This then begs the question, why do these species with a higher ASP have a lower CTN? This seeming paradox may be partially explained by the similar expression levels of genes along all four ASP/L-related pathways, or the smaller differences in expression between genes along the spliceosome pathway and the other three ASP/L-related KEGG pathways in Hvu and Tca compared with those observed for species with a higher OC (Additional file 14: Figure S7A). Therefore, although Hvu has a higher ASP that is attributable to the higher expression of genes along the spliceosome pathway, most of its transcripts are degraded by the mRNA surveillance and RNA degradation pathways. Consequently, only a fraction of its transcripts is translated into proteins. Our findings that the brain and testis have the highest ASP/L is consistent with a previous study performed across three mammals [34], which indicates a conserved phenomenon within the Metazoa.

ASP/L displays a positive correlation with mean intron size and intron density and is negatively correlated with CDS density among genes within individual genomes. The underlying mechanisms of these results are still being revealed. It is reported that exon definition, which tends to occur on cassette exons flanked by longer introns, is less efficient than intron definition, which tends to occur on constitutive exons flanked by shorter introns [11]. Several studies have found that exons flanked by longer introns tend to have a higher density of exonic splicing enhancers (ESEs) at both ends [35–37] and strong splice sites [36, 38], which facilitate effective exon splicing. Longer introns also harbor more non-conserved splicing sites, which occur in the vicinity of regions enriched in genomic features that define exons [39]. This result suggests that longer introns tend to produce more usable transcripts.

The next question then is why do the mechanisms that produce higher ASP/L for longer introns within individual genomes not work across species? Previous studies have found that the cis-acting splicing regulatory elements, including ESEs and splice sites, are conserved within vertebrates [37, 38]. Splicing-related genomic features, such as the number of U12 introns, of a large genome from an invertebrate are more similar to those of vertebrates than to those of small genomes from invertebrates [13]. Therefore, we speculate that the trans-regulatory factors interacting with other ASP/L-related proteins are responsible for this regulation, as discussed below.

In our study, the four ASP/L-related pathways contributed to the ASP/L differences across the species with different OC. In particular, the spliceosome pathway, which is comprised of splicing factors, displayed distinct genomic features, including the highest expression level, smallest evolutionary distance, and highest fraction of disordered regions across all species. Furthermore, the spliceosome pathway displayed the most significant correlations between OC and ASP/L, the fraction of disordered regions, and the fraction of disordered binding regions. These results suggested that (1) the relationships between the spliceosome and the other three pathways with respect to these features are conserved across Metazoan species, and (2) the spliceosome pathway may play a more important role than the other three pathways in the evolution of OC. Several studies have reported that various phenotypes and diseases are regulated by splicing factors through their varying expression level, copy number, amino acid mutation, and number of isoforms among individuals from the same species or among multiple species [28, 40–43]. In line with these findings, we revealed direct correlations between OC and various genomic features of splicing factors. Several of these features, such as gene copy number, ASP/L, the fraction of protein disordered region, and the fraction consisting of the protein disordered binding sites, have been reported to be associated with OC with regard to the protein-coding genes in the genome [3, 8, 17] or specific groups, such as transcription factors [18, 19]. These genomic features are capable of expanding the number of distinct proteins with an unchanged gene number and an increasing number of interacting proteins, which, in turn, contributes to increasing OC [3]. Our finding of a positive correlation between the evolutionary distance of splicing factor proteins and OC suggests that variations at the sequence level of splicing factors also contribute to OC [44, 45]. To the best of our knowledge, this is the first report of a direct correlation between OC and splicing factors.

Although the six genomic features were positively correlated with OC, only weak or no correlations were found among them. This result indicates that these features may independently promote ASP/L through specific means. Thus, from a network perspective, these features likely impact distinct parts of the network [46]. Increasing the level of gene expression means intensifying a single node, whereas a higher ASP/L and gene copy number mean that more nodes will be present in the network. Furthermore, a lower evolutionary distance implies a higher functional similarity among nodes within the networks from different species, whereas the disordered region and disordered binding site expansion produce more edges in the network. We thus depicted our findings as Fig. 6 C. It may through the cooperated six genomic features, splicing-related proteins increase organism’s splicing level, which further promote the organism complexity to a higher level.

Although we included as many species as possible in this study, many more species were excluded for various reasons. Besides the above-mentioned exclusion of low-quality genome assemblies and gene annotations along with inadequate RNA-Seq data, the main reason for species exclusion was a lack of CTN information. There are currently only 42 orders that have at least one species with known CTNs, and there are only 30 unique CTNs [3]. Therefore, more publicly available high-quality annotated genomes and additional RNA-Seq data from various tissues and developmental stages are required for future studies, particularly data from species with known CTNs.

## Conclusions

Our results suggest that the positive correlation between ASP/L and OC ubiquitously exists in eukaryotes, and this correlation is not affected by the mean intron size of these species. However, within a single genome, we showed that ASP/L was indeed affected by the mean intron size. We further demonstrated that splicing factors may improve OC through gradually changing several genomic features.

## Methods

### Datasets used

The FirstSpeciesSet comprised 14 species with a genome size equal to or greater than that of the human genome for which the full genome sequence, gene annotations, and at least 19 Gb RNA-Seq data were available. These species included 11 from Deuterostomia (*Homo sapiens*, *Pan troglodytes*, *Dasypus novemcinctus*, *Monodelphis domestica*, *Ambystoma mexicanum*, *Leptobrachium leishanense*, *Microcaecilia unicolor*, *Scyliorhinus torazame*, *Chiloscyllium punctatum*, *Erpetoichthys calabaricus*, and *Sphenodon punctatus*) and three from Protostomia (*Locusta migratoria*, *Procambarus virginalis*, and *Euprymna scolopes*) (Additional file 1: Table S1.1). The SecondSpeciesSet comprised 14 species, which included 12 from Bilateria (*Toxocara canis*, *Amphibalanus amphitrite*, *Penaeus vannamei*, *Drosophila pseudoobscura*, *Glossina pallidipes*, *Drosophila simulans*, *Ciona savignyi*, *Branchiostoma floridae*, *Takifugu rubripes*, *Cyclopterus lumpus*, *Anarrhichthys ocellatus*, and *Seriola lalandi*) and two from Cnidaria (*Orbicella faveolata* and *Hydra vulgaris*) (Additional file 1: Table S1.2). The ThirdSpeciesSet comprised 12 dipteran species, which included five from Culicidae (*Culex quinquefasciatus*, *Aedes aegypti*, *Aedes albopictus*, *Anopheles albimanus*, *Anopheles gambiae*), three from Drosophilidae (*Drosophila melanogaster*, *D. simulans*, *D. pseudoobscura*), two from *Muscidae* (*Musca domestica*, *Stomoxys calcitrans*), and one each from Chironomidae (*Parochlus steinenii*) and Glossinidae (*Glossina pallidipes*) (Additional file 1: Table S1.3). The quality of the protein sets used for each species was evaluated using BUSCO (v10) [47]. The parameter “-l metazoa” was used for the First- and SecondSpeciesSet, and “-l diptera” was used for the ThirdSpeciesSet.

OC data measured based on CTN were from [3]. For the species without CTN data, the CTN information from the most closely related species was used instead. Genome sequence and gene annotation data were downloaded from the US National Center for Biotechnology Information (NCBI) Genome database (https://www.ncbi.nlm.nih.gov/genome). RNA-Seq data for each species were downloaded from the NCBI Sequence Read Archive (SRA) database (https://www.ncbi.nlm.nih.gov/sra) and the European Bioinformatics Institute (EBI) ArrayExpress database (https://www.ebi.ac.uk/arrayexpress). For the study with “selection method” denoted as “RANDOM” or “unspecified”, we manually confirmed their selection method as “Oligo-dT” through reading the published literature that cited the corresponding data set, or the description of the entry in NCBI/SRA database. The datasets used are summarized in Additional file 2: Table S2.

### Construction of six RNA-Seq datasets

To test the impact of sequencing depth and tissue type on alternative splicing detection, we constructed four RNA-Seq datasets derived from the FirstSpeciesSet, D1 to D4. D1 consisted of all available data for each species with a minimum of 19 Gb data. By using a binning method with 500 genes in each bin, we found that there were at least four bins with unchanged ASP/L, as predicted by the fitted sigmoid curve, which indicates that the sequencing depth was saturated (Additional file 3: Figure S1). D2 was comprised of 100 million reads with an equal length of 75 bp that were randomly sampled from total reads for each species. This dataset allowed us to test the splicing level among species with equivalent data quantities. D3 included all available data for four tissue types (brain, liver, testis, and kidney) for 12 species (two species had data from fewer than four tissues and were excluded from this dataset; Additional file 5: Table S4). The D3 dataset allowed us to test ASP/L with the same number and types of tissues. D4 consisted of 12 million reads that were randomly sampled from data for each of the four tissues and across all 12 species in D3 so that a total of 48 million reads were included.

To test whether the positive correlation between ASP/L and OC still existed on the single-tissue level, we designed another two data sets, D5 and D6. D5 contained all of the available data from five tissue types (brain, liver, kidney, testis, and muscle) for which at least six species had RNA-Seq data. D6 included equal quantities of data sampled randomly across all species for each of the five tissues in D5 (Additional file 7: Table S6).

### Measurement of ASP/L

To identify isoforms/splicing events, we chose three widely used and representative tools: LeafCutter [24], StringTie [48], and SpliceTrap [49]. These tools use different quantification strategies: junction-level (LeafCutter), isoform-level (StringTie), and exon-level (SpliceTrap). None of these strategies are completely accurate [50, 51]. ASP/L was calculated based on all expressed multi-exon genes and using an expression level rank-based binning method with either 100 or 500 genes in each bin (Additional file 3: Figure S1).

To run LeafCutter, we first aligned the raw RNA-Seq raw reads to the corresponding reference genome sequence using HISAT2 [52] with default parameters. The unique mapped reads were used to produce junctions with the *bam2junc.sh* script in LeafCutter [24]. Multiple junction files for each species were merged and the number of reads supporting the junctions was summed. The *leafcutter_cluster.py* script was used to cluster the junctions and produce the intron clusters with the parameters ‘-l 500000 -m 30’. We filtered the intron clusters that connected more than one reference gene. The coverage depth of each species was calculated using *bedtools genomecov* with the parameters ‘-bg -split’ for each sample and then merged using *bedtools unionbedg*. The expression level was approximated as the maximum coverage depth in the gene exon regions. Multi-exon genes were considered expressed if their exon regions had at least one bp length with a coverage depth > 2. ASP was defined as the percentage of expressed multi-exon genes with at least one intron cluster. We used *Shannon’s entropy* to measure ASP/L [53]. For each intron cluster with *m* junctions, each junction was connected by *n*_*k*_ reads, such that the percentage of reads that supported the *k* junction was ${f}_{k}= \frac{{n}_{k}}{{\sum }_{k=1}^{m}{n}_{k}}$. We then calculated the *Shannon’s entropy* of the intron cluster as $H= -\sum _{k=1}^{m}{f}_{k}{log}_{2}{f}_{k}$ and summed the entropy from all intron clusters of each gene as the gene-level ASL.

With the StringTie tool, we first ran StringTie (v1.3.4) for each sample with the parameter ‘-j 3’ and then merged the multiple gtf files using “stringtie --merge” with the parameters ‘-c 0.01 -F 1 -T 1 -f 0.01’. We only used the genes that were *de novo* annotated by StringTie and overlapped with genes from Official Gene Set for alternative splicing analysis. We calculated the gene expression level as the Fragments Per Kilobase of transcript per Million mapped reads (FPKM) using StringTie with the parameters “-b -e -G” for each sample. For the transcripts from each gene, we summed their expression levels across samples to determine the gene expression level. ASP was calculated as the percentage of expressed multi-exon genes (FPKM > 0.1) with more than one isoform. ASL was calculated as the mean number of isoforms for each expressed multi-exon gene.

With the SpliceTrap tool, we first created pseudochromosomes comprised of a maximum of 30 chromosomes and concatenating scaffolds with 100 ‘N’s. We used the merged gtf files generated by StringTie using all available data for each species as the reference to create a TXdb database using the *TXdbgen* scripts in SpliceTrap. SpliceTrap was run using the Bowtie aligner without Inclusion-Ratio distribution Model correction for each sample. We merged the ratio files from all samples for each species by summing the number of junction reads across all samples. The *apply_cutoff.sh* script was used to filter the data in the final ratio result file with the cutoffs of medium stringency and at least five junction reads. The gene expression level was calculated using StringTie. The ASP was calculated as the percentage of expressed multi-exon genes (FPKM > 0.1) with more than one splicing event. ASL was calculated as the mean number of events for each multi-exon gene.

### Influence of sequencing depth on ASP/L

To simulate the influence of sequencing depth on ASP/L, we used a previously published method [23] to split the expressed multi-exon genes into bins of the same size according to their expression level. For each bin, we calculated the ASP/L. A sigmoid curve was then fitted to the function$F\left(x\right)=\frac{\alpha }{1+{e}^{1\left(x-\alpha \right)*b}}$ for ASP/L (*F(x)*) in each bin as a function of gene expression (*x*), where α, the upper asymptote, represented the true extent of ASP/L. The sigmoidal model was computed using nls in the basic *stats* package of R and SSlogis function. In contrast to the results of Wang et al. [23], we discovered a decreasing trend in ASP/L when the sequencing depth or expression level was greater than a certain level. Therefore, in addition to the estimated upper asymptote, we also calculated the mean ASP/L values of the bins with a predicted value variation of < 0.0001.

For the binning size selection, Wang et al. [23] have found that the standard deviation (SD) of ASP is 0.01 when the binning size varied from 50 to 500 with step 50. However, because the data amount and species used in this study are very different from Wang’s study, we re-evaluated the robustness of binning size using the same binning size set as Wang’s. The evaluation was performed using the D1 to D4 dataset from the FirstSpeciesSet and all three isoform/event identification software as Fig. 2 A illustrated. For each combination of the dataset, software, and species, we calculated the ASP/L at ten binning size levels, then calculated the SD of the ASP/L. Totally, 520 SD values were obtained, half of them are ASL and ASP, respectively. We found 95% of these SD values from ASP are less than 0.007, this value for ASL is 0.06. We also calculated the Spearman’s correlation coefficient between ASP/L and OC and then the SD for each combination of the dataset and software. Totally, 40 SD values were obtained, and 95% of these SD values are less than 0.03. These results suggest that the binning size used in this study is robust and the correlation results in this study is not influenced by the binning size. We therefore selected two bin sizes, 500 and 100, to calculate the ASP/L in this study.

### Correlation calculations

To measure the correlation between ASP/L and OC, we used three methods, i.e., Spearman’s correlation, linear regression, and PGLS regression. The first two methods were performed using the *cor* and *lm* functions in the R stats package. The PGLS regression was performed using the *pgls* function in the R caper package. The *λ* parameter was estimated using the maximum likelihood method. The phylogenetic trees were constructed as described below.

### Species phylogenetic tree construction

To construct a phylogenetic tree of the FirstSpeciesSet, we used *Dendronephthya gigantea* (GCF_004324835.1, DenGig_1.0) as the outgroup. We retained the longest isoforms for genes with multiple isoforms. The OrthoFinder (v2.3.11) package [54] was used to construct the phylogenetic tree of the 15 species with the parameters ‘-S diamond -T fasttree’. The rooted species tree was inferred using STRIDE [55], which is embedded in OrthoFinder. The same method was used to construct a phylogenetic tree of the SecondSpeciesSet, which included 14 species and *Amphimedon queenslandica* as the outgroup, and the ThirdSpeciesSet, which included 12 dipteran species and *Bombyx mori* as the outgroup.

### KEGG pathway-based analysis

Genome-wide KEGG pathway annotation was performed using the webserver GhostKOALA [56]. The expression levels of the ASP/L-related genes were calculated as quantiles, with larger quantiles representing higher expression. The evolutionary distance calculations based on the phylogenetic species trees were performed using the *cophenetic.phylo* function in the R ape package [57]. For each KO, we first performed multiple sequence alignment using MUSCLE (v3.8.31) [58] and then calculated the evolutionary distance using the *dist.alignment* function in the R seqinr package [59]. Disordered residues and disordered binding residues were predicted using the IUPred2A tool [60]. Residues were considered disordered residues or disordered binding residues if their prediction score was > 0.5.

### Visualization and statistics

ComplexHeatmap [61] was used to visualize the *P* value matrix (Fig. 2 A) and set interaction (Fig. 6 A). Principal component analysis was performed using the R ggfortify package [62].

## Supplementary Information

**Additional file 1: Table S1.** Summary of the three sets of species used in this study.

**Additional file 2: Table S2.** Details of the RNA-Seq datasets used in this study.

**Additional file 3: Figure S1.** Distribution of alternative splicing prevalence. This analysis was performed using dataset D1, the LeafCutter tool, and 500 genes in each bin. The black line was plotted using real data, whereas the red line was simulated using the sigmoid function. AS, alternative splicing.

**Additional file 4: Table S3.** Summary statistics of the RNA-Seq data from the FirstSpeciesSet.

**Additional file 5: Table S4.** The four selected tissues used for each species in the FirstSpeciesSet.

**Additional file 6: Table S5.** ASP/L and correlation with the cell type number (CTN) and mean intron size for the three sets of species.

**Additional file 7: Table S6.** Statistics of the five tissues used for tissue-level ASP/L calculation for the FirstSpeciesSet.

**Additional file 8: Figure S2.** ASP/L displayed a positive correlation with organism complexity when using the D5 and D6 datasets. The boxplot indicates the P value with -log10 transformation for each column. PASP, predicted alternative splicing prevalence; ASP, actual alternative splicing prevalence; PASL, predicted alternative splicing level; ASL, actual alternative splicing level; GeneNum, gene number; SplicLvl, splicing level; CorMethod, correlation calculation method.

**Additional file 9: Table S7.** The two tissues with the highest ASP/L in the FirstSpeciesSet.

**Additional file 10: Figure S3.** Distribution of alternative splicing prevalence and level (ASP/L) against cell type number (CTN) and mean intron size across all 37 species used in this study. (A) The red points represent three outlier species that have higher ASP but lower CTN. ASP was calculated using all RNA-Seq data for each species, LeafCutter software, and the Bin 500 method. Spearman’s ρ = 0.63, P = 1.27e-05. (B) The scatter plots depict the correlation between mean intron size and two species-level complexity proxies, organism complexity (CTN; the blue points and the y-axis on the left) and alternative splicing level (ASL; the red points and the y-axis on the right). ASL was calculated using the LeafCutter tool and a Bin500 gene number. Spearman’s ρ between mean intron size and CTN is 0.36 (P = 0.02), and between mean intron size and ASL is 0.46 (P = 0.0026).

**Additional file 11: Figure S4.** Distribution of Spearman’s ρ between three intron size-related statistics and cell type number (CTN) and alternative splicing level (ASL) when using the data from Chen et al. (2014), the FirstSpeciesSet, and the SecondSpeciesSet.

**Additional file 12: Figure S5.** Influence of highly expressed genes on the calculation of correlations between ASP/L and intron size-related statistics across all three sets of species. The analysis details are the same as those described in the figure legend for Fig. 3E. The green boxes represent the correlations or P values calculated using highly expressed genes, whereas the purple boxes represent those values calculated using genes with all genes. Although the correlations and P values varied between the highly expressed genes and all genes, these differences were not significant. MeanIntr, mean intron size; CDSdens, coding sequence density; IntrDens, intron density.

**Additional file 13: Figure S6.** Species with higher organism complexity had higher expression-level differences between genes along the spliceosome pathway (ko03040) and the other three mRNA biogenesis and transport pathways (ko03013, ko03025, and ko03018). (A, B) StringTie analysis using the expression data from the D1 dataset. (C, D) LeafCutter analysis using the D2 dataset. (A, C) Expression-level distribution of ASP/L-related pathways and organism complexity. (B, D) Positive correlation between the Wilcoxon W-statistics and organism complexity (represented by cell type number, CTN) (D1_StringTie, Spearman’s ρ = 0.63, P = 0.015; D2_LeafCutter, ρ = 0.62, P = 0.017). The Wilcoxon rank-sum test was used to calculate the W-statistic between the spliceosome pathway (ko03040) and the other three mRNA biogenesis and transport pathways (ko03013, ko03025, and ko03018).

**Additional file 14: Figure S7.** Relationships between organism complexity and the six genomic features of splicing factors in the SecondSpeciesSet. The analysis details are the same as those described in the figure legend of Fig. 5. The four ASP/L-related pathways investigated were RNA transport (ko03013), mRNA surveillance pathway (ko03015), RNA degradation (ko03018), and spliceosome (ko03040). The gene expression levels were calculated using LeafCutter (A), and, for each species, the Wilcoxon W-statistics was calculated between ko03040 and the combination of the other three pathways (B), which yielded Spearman’s ρ = 0.39, P = 0.17. (C) Scatter plots of the genome-wide protein-coding genes (left) and those along the four pathways (right) vs. the cell type number (CTN). The gene numbers in the four pathways were positively correlated with organism complexity using the genome-wide protein-coding genes as the background (Chi-square test: ko03040, ko03013, ko03018, and ko03015, P = 5.8e-26, 6.9e-15, 6.3e-07, and 7.7e-12, respectively). (D) Scatter plots of the evolutionary distance and organism complexity differences across the 14 species based on the species phylogenetic tree and the protein alignment of the KEGG orthologs along the four pathways (right) vs. the CTN difference. The CTN difference was calculated as diff(a,b)/max(a,b) and ranged between 0 and 1. The evolutionary distances were positively correlated with the organism complexity differences (left: Spearman’s ρ = 0.4, P = 6.5e-05; right: for ko03040, ko03013, ko03015, and ko03018, ρ = 0.49, 0.43, 0.44, and 0.44, respectively, and P = 1.4e-04, 1.2e-03, 7.8e-04, and 8.5e-04, respectively). (E) Positive correlations between ASP and CTN using the LeafCutter tool for all genes (left) and the genes along the four pathways (right) (left: Spearman’s ρ = 0.66, P = 0.03; right: for ko03013, ko03015, ko03018, and ko03040, Spearman’s ρ = 0.54, 0.5, 0.42, and 0.61, respectively, and P = 0.09, 0.11, 0.2, and 0.05, respectively). (F) Positive correlations between the fraction of disordered residues and CTN for all genes (left: Spearman’s ρ = 0.31, P = 0.28) and genes along the four pathways (right: for ko03013, ko03015, ko03018 and ko03040, Spearman’s ρ = 0.38, 0.3, 0.52, and 0.58, respectively, and P = 0.19, 0.31, 0.06, and 0.03, respectively). (G) Scatter plots of the fraction of binding residues vs. CTN for all genes (left: Spearman’s ρ = 0.3, P = 0.3) and genes along the four pathways (right: for ko03013, ko03015, ko03018 and ko03040, Spearman’s ρ = 0.55, 0.38, 0.62, and 0.52, respectively, and P = 0.04, 0.19, 0.02, and 0.06, respectively).

**Additional file 15: Table S8.** Correlation between organism complexity and the six genomic features of ASP/L-related pathways in the FirstSpeciesSet.

**Additional file 16: Figure S8.** Correlation among genomic features of ASP/L-related genes in the SecondSpeciesSet. (A) Intersection of significant KOs among six genomic features. Spearman correlation and PGLS regression were calculated between these genomic features and organism complexity for each KO in the SecondSpeciesSet. Significant KOs were defined as Spearman’s ρ > 0.4 and PGLS P < 0.05. (B) Principal component analysis of these six genomic features based on the Spearman’s ρ in 112 KOs. The points represent KOs and the shapes represent the four pathways.

## Data Availability

All the sequencing data used here (listed in Additional file 2) have been published previously and can be found in NCBI Sequence Read Archive database (https://www.ncbi.nlm.nih.gov/sra).

## Abbreviations

RNA-Seq: RNA-sequencing
ASL: alternative splicing level
ASP: alternative splicing prevalence
ASP/L: alternative splicing prevalence and level
CTN: cell type number
OC: organism complexity
PGLS: phylogenetic generalized least squares
PASP: predicted alternative splicing prevalence
PASL: predicted alternative splicing level
KEGG: Kyoto Encyclopedia of Genes and Genomes
KO: KEGG Orthology
