# Supplementary material for: Alternative splicing level related to intron size and organism complexity
Source: BMC Genomics. 2021 Nov 25;22:853. doi: 10.1186/s12864-021-08172-2 (PMC8614042; doi:10.1186/s12864-021-08172-2)
Supplement: Supplementary file 3 — Additional file 3: Figure S1. Distribution of alternative splicing prevalence. This analysis was performed using dataset D1, the LeafCutter tool, and 500 genes in each bin. The black line was plotted using real data, whereas the red line was simulated using the sigmoid function. AS, alternative splicing. [file 12864_2021_8172_MOESM3_ESM.pdf]

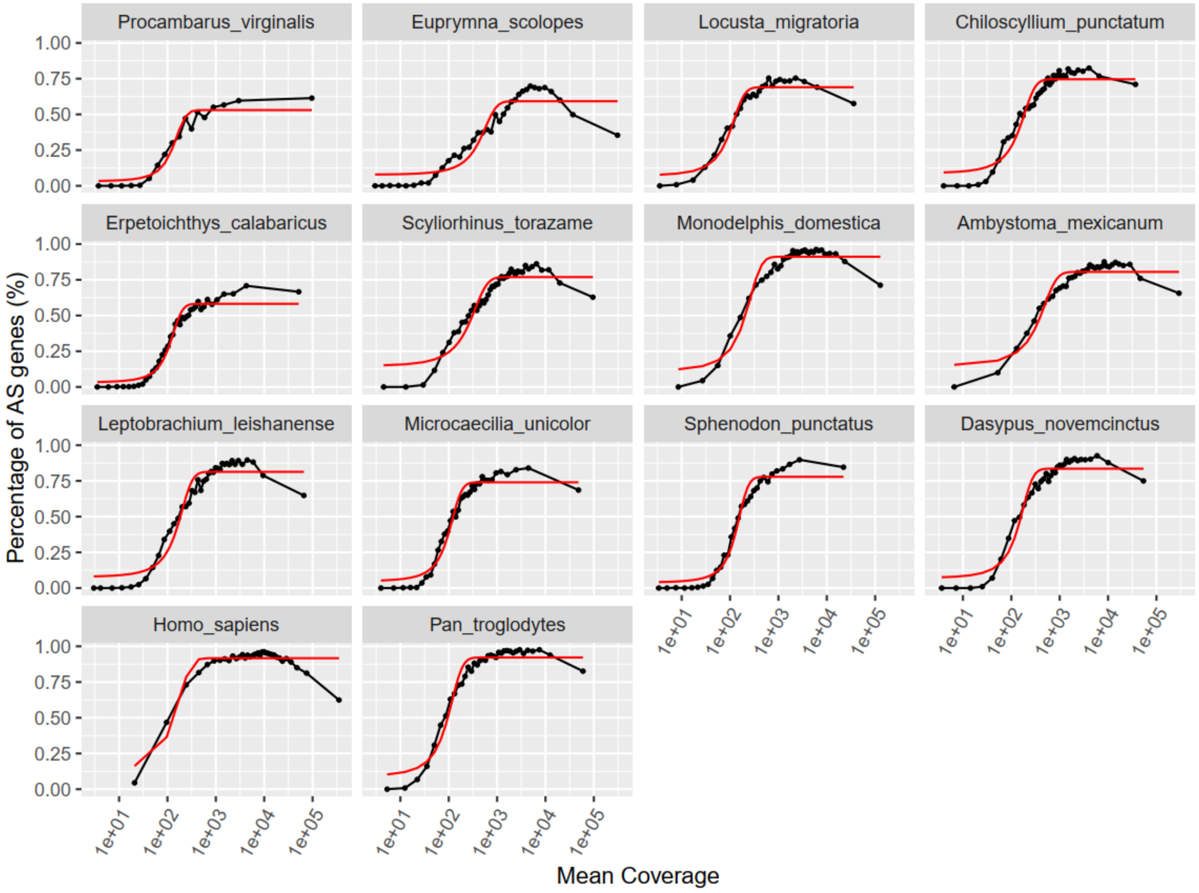

Supplementary Fig. S1. Distribution of alternative splicing prevalence. This analysis was performed using dataset D1, the LeafCutter tool, and 500 genes in each bin. The black line was plotted using real data, whereas the red line was simulated using the sigmoid function. AS, alternative splicing.
