# Supplementary material for: Alternative splicing level related to intron size and organism complexity
Source: BMC Genomics. 2021 Nov 25;22:853. doi: 10.1186/s12864-021-08172-2 (PMC8614042; doi:10.1186/s12864-021-08172-2)
Supplement: Supplementary file 10 — Additional file 10: Figure S3. Distribution of alternative splicing prevalence and level (ASP/L) against cell type number (CTN) and mean intron size across all 37 species used in this study. (A) The red points represent three outlier species that have higher ASP but lower CTN. ASP was calculated using all RNA-Seq data for each species, LeafCutter software, and the Bin 500 method. Spearman’s ρ = 0.63, P = 1.27e-05. (B) The scatter plots depict the correlation between mean intron size and two species-level complexity proxies, organism complexity (CTN; the blue points and the y-axis on the left) and alternative splicing level (ASL; the red points and the y-axis on the right). ASL was calculated using the LeafCutter tool and a Bin500 gene number. Spearman’s ρ between mean intron size and CTN is 0.36 (P = 0.02), and between mean intron size and ASL is 0.46 (P = 0.0026). [file 12864_2021_8172_MOESM10_ESM.pdf]

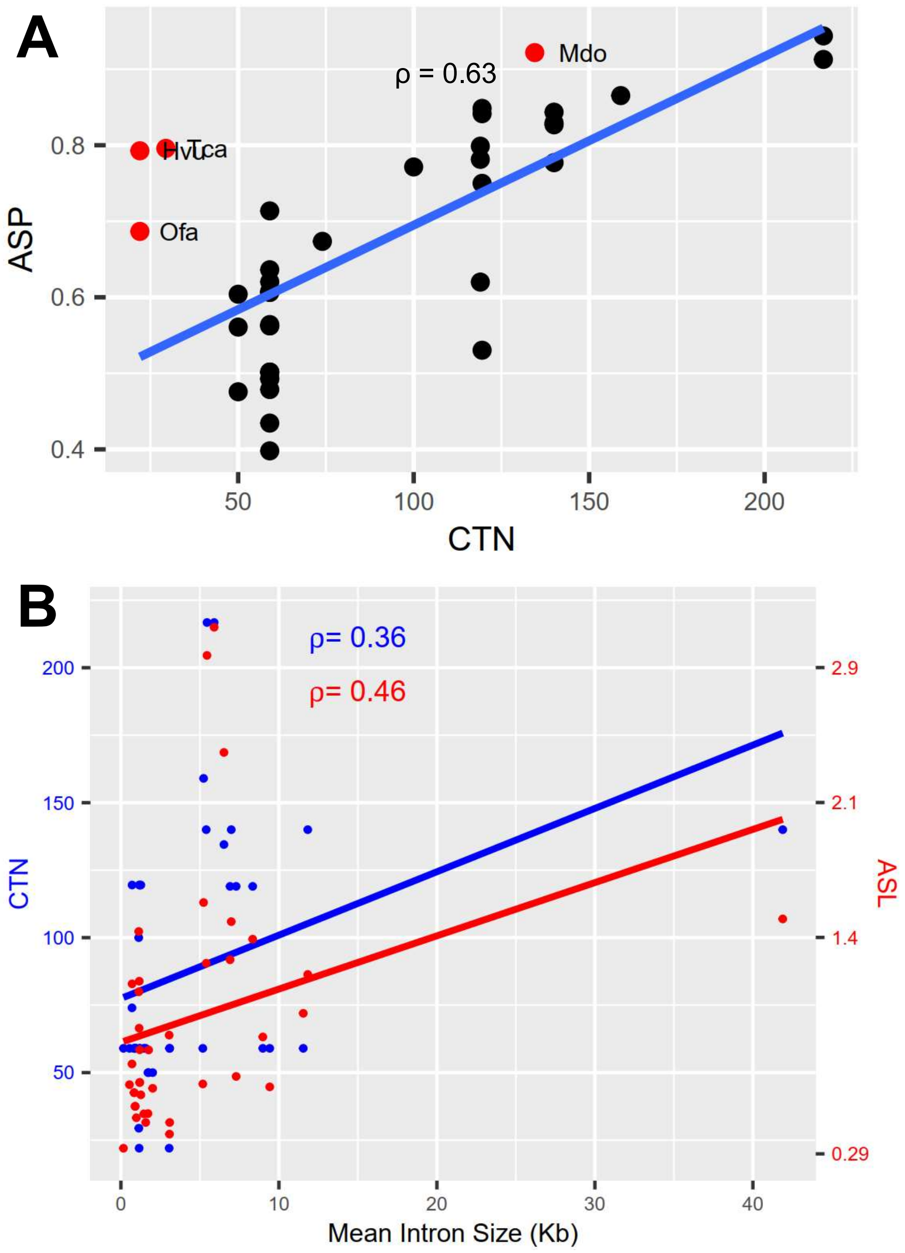

Supplementary Fig. S3. Distribution of alternative splicing prevalence and level (ASP/L) against cell type number (CTN) and mean intron size across all 37 species used in this study. (A) The red points represent three outlier species that have higher ASP but lower CTN. ASP was calculated using all RNA-Seq data for each species, LeafCutter software, and the Bin 500 method. Spearman's ρ = 0.63, *P* = 1.27e-05. (B) The scatter plots depict the correlation between mean intron size and two species-level complexity proxies, organism complexity (CTN; the blue points and the y-axis on the left) and alternative splicing level (ASL; the red points and the y-axis on the right). ASL was calculated using the LeafCutter tool and a Bin500 gene number.
