# Supplementary material for: Alternative splicing level related to intron size and organism complexity
Source: BMC Genomics. 2021 Nov 25;22:853. doi: 10.1186/s12864-021-08172-2 (PMC8614042; doi:10.1186/s12864-021-08172-2)
Supplement: Supplementary file 11 — Additional file 11: Figure S4. Distribution of Spearman’s ρ between three intron size-related statistics and cell type number (CTN) and alternative splicing level (ASL) when using the data from Chen et al. (2014), the FirstSpeciesSet, and the SecondSpeciesSet. [file 12864_2021_8172_MOESM11_ESM.pdf]

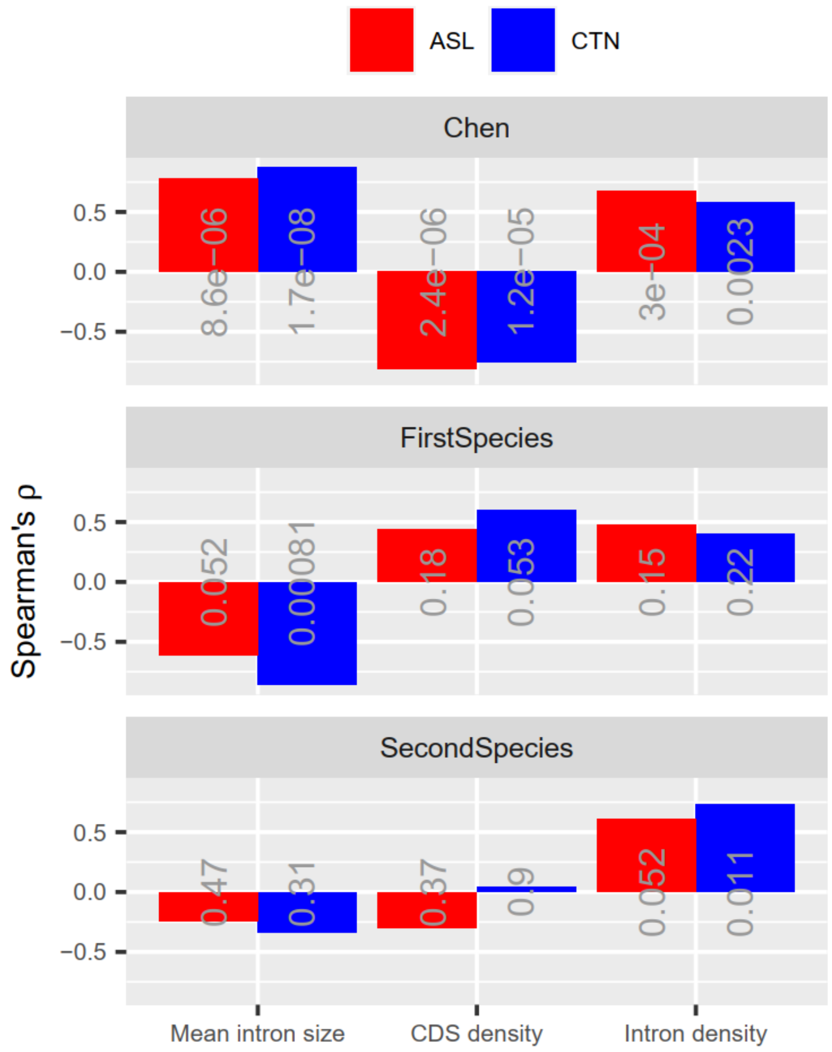

Supplementary Fig. S4. Distribution of Spearman's ρ between three intron size-related statistics and cell type number (CTN) and alternative splicing level (ASL) when using the data from Chen et al. (2014), the FirstSpeciesSet, and the SecondSpeciesSet.
