# Supplementary material for: Alternative splicing level related to intron size and organism complexity
Source: BMC Genomics. 2021 Nov 25;22:853. doi: 10.1186/s12864-021-08172-2 (PMC8614042; doi:10.1186/s12864-021-08172-2)
Supplement: Supplementary file 12 — Additional file 12: Figure S5. Influence of highly expressed genes on the calculation of correlations between ASP/L and intron size-related statistics across all three sets of species. The analysis details are the same as those described in the figure legend for Fig. 3E. The green boxes represent the correlations or P values calculated using highly expressed genes, whereas the purple boxes represent those values calculated using genes with all genes. Although the correlations and P values varied between the highly expressed genes and all genes, these differences were not significant. MeanIntr, mean intron size; CDSdens, coding sequence density; IntrDens, intron density. [file 12864_2021_8172_MOESM12_ESM.pdf]

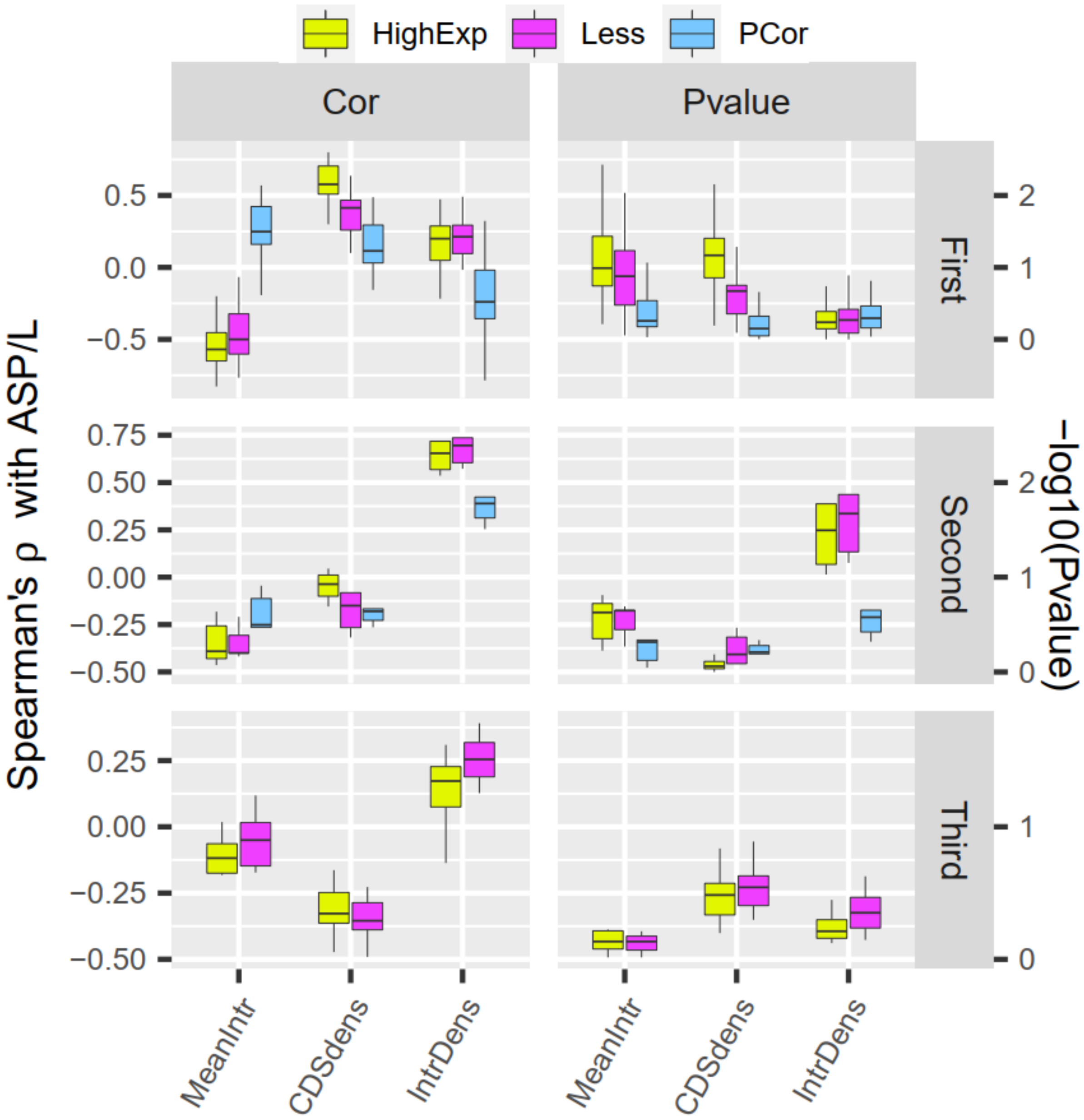

Supplementary Fig. S5. Influence of highly expressed genes on the calculation of correlations between ASP/L and intron size-related statistics across all three sets of species. The analysis details are the same as those described in the figure legend for Fig. 3E. The green boxes represent the correlations or *P* values calculated using highly expressed genes, whereas the purple boxes represent those values calculated using genes with all genes. Although the correlations and *P* values varied between the highly expressed genes and all genes, these differences were not significant. MeanIntr, mean intron size; CDSdens, coding sequence density; IntrDens, intron density.
