# Supplementary material for: Alternative splicing level related to intron size and organism complexity
Source: BMC Genomics. 2021 Nov 25;22:853. doi: 10.1186/s12864-021-08172-2 (PMC8614042; doi:10.1186/s12864-021-08172-2)
Supplement: Supplementary file 13 — Additional file 13: Figure S6. Species with higher organism complexity had higher expression-level differences between genes along the spliceosome pathway (ko03040) and the other three mRNA biogenesis and transport pathways (ko03013, ko03025, and ko03018). (A, B) StringTie analysis using the expression data from the D1 dataset. (C, D) LeafCutter analysis using the D2 dataset. (A, C) Expression-level distribution of ASP/L-related pathways and organism complexity. (B, D) Positive correlation between the Wilcoxon W-statistics and organism complexity (represented by cell type number, CTN) (D1_StringTie, Spearman’s ρ = 0.63, P = 0.015; D2_LeafCutter, ρ = 0.62, P = 0.017). The Wilcoxon rank-sum test was used to calculate the W-statistic between the spliceosome pathway (ko03040) and the other three mRNA biogenesis and transport pathways (ko03013, ko03025, and ko03018). [file 12864_2021_8172_MOESM13_ESM.pdf]

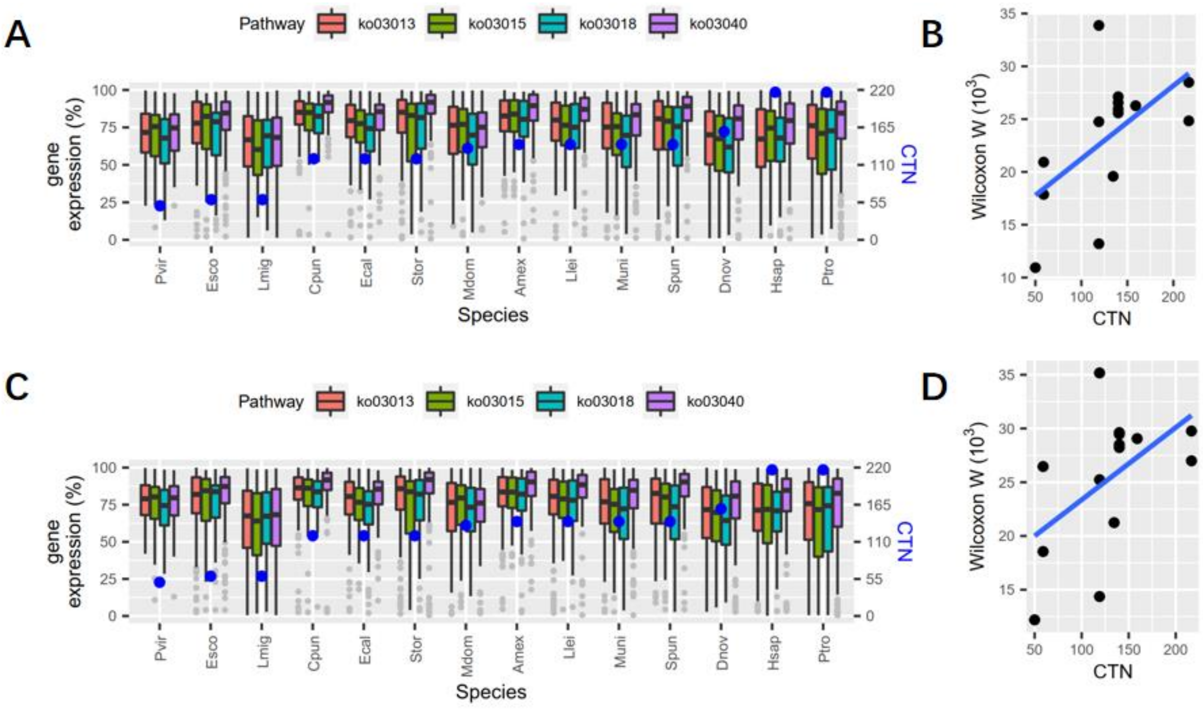

Supplementary Fig. S6. Species with higher organism complexity had higher expression-level differences between genes along the spliceosome pathway (ko03040) and the other three mRNA biogenesis and transport pathways (ko03013, ko03025, and ko03018). (A, B) StringTie analysis using the expression data from the D1 dataset. (C, D) LeafCutter analysis using the D2 dataset. (A, C) Expression-level distribution of ASP/L-related pathways and organism complexity. (B, D) Positive correlation between the Wilcoxon W-statistics and organism complexity (represented by cell type number, CTN) (D1_StringTie, Spearman's ρ= 0.63, P = 0.015; D2_LeafCutter, ρ = 0.62, P = 0.017). The Wilcoxon rank-sum test was used to calculate the W-statistic between the spliceosome pathway (ko03040) and the other three mRNA biogenesis and transport pathways (ko03013, ko03025, and ko03018).
