# Supplementary material for: Alternative splicing level related to intron size and organism complexity
Source: BMC Genomics. 2021 Nov 25;22:853. doi: 10.1186/s12864-021-08172-2 (PMC8614042; doi:10.1186/s12864-021-08172-2)
Supplement: Supplementary file 14 — Additional file 14: Figure S7. Relationships between organism complexity and the six genomic features of splicing factors in the SecondSpeciesSet. The analysis details are the same as those described in the figure legend of Fig. 5. The four ASP/L-related pathways investigated were RNA transport (ko03013), mRNA surveillance pathway (ko03015), RNA degradation (ko03018), and spliceosome (ko03040). The gene expression levels were calculated using LeafCutter (A), and, for each species, the Wilcoxon W-statistics was calculated between ko03040 and the combination of the other three pathways (B), which yielded Spearman’s ρ = 0.39, P = 0.17. (C) Scatter plots of the genome-wide protein-coding genes (left) and those along the four pathways (right) vs. the cell type number (CTN). The gene numbers in the four pathways were positively correlated with organism complexity using the genome-wide protein-coding genes as the background (Chi-square test: ko03040, ko03013, ko03018, and ko03015, P = 5.8e-26, 6.9e-15, 6.3e-07, and 7.7e-12, respectively). (D) Scatter plots of the evolutionary distance and organism complexity differences across the 14 species based on the species phylogenetic tree and the protein alignment of the KEGG orthologs along the four pathways (right) vs. the CTN difference. The CTN difference was calculated as diff(a,b)/max(a,b) and ranged between 0 and 1. The evolutionary distances were positively correlated with the organism complexity differences (left: Spearman’s ρ = 0.4, P = 6.5e-05; right: for ko03040, ko03013, ko03015, and ko03018, ρ = 0.49, 0.43, 0.44, and 0.44, respectively, and P = 1.4e-04, 1.2e-03, 7.8e-04, and 8.5e-04, respectively). (E) Positive correlations between ASP and CTN using the LeafCutter tool for all genes (left) and the genes along the four pathways (right) (left: Spearman’s ρ = 0.66, P = 0.03; right: for ko03013, ko03015, ko03018, and ko03040, Spearman’s ρ = 0.54, 0.5, 0.42, and 0.61, respectively, and P = 0.09, 0.11, 0.2, and 0.05, respec [file 12864_2021_8172_MOESM14_ESM.pdf]

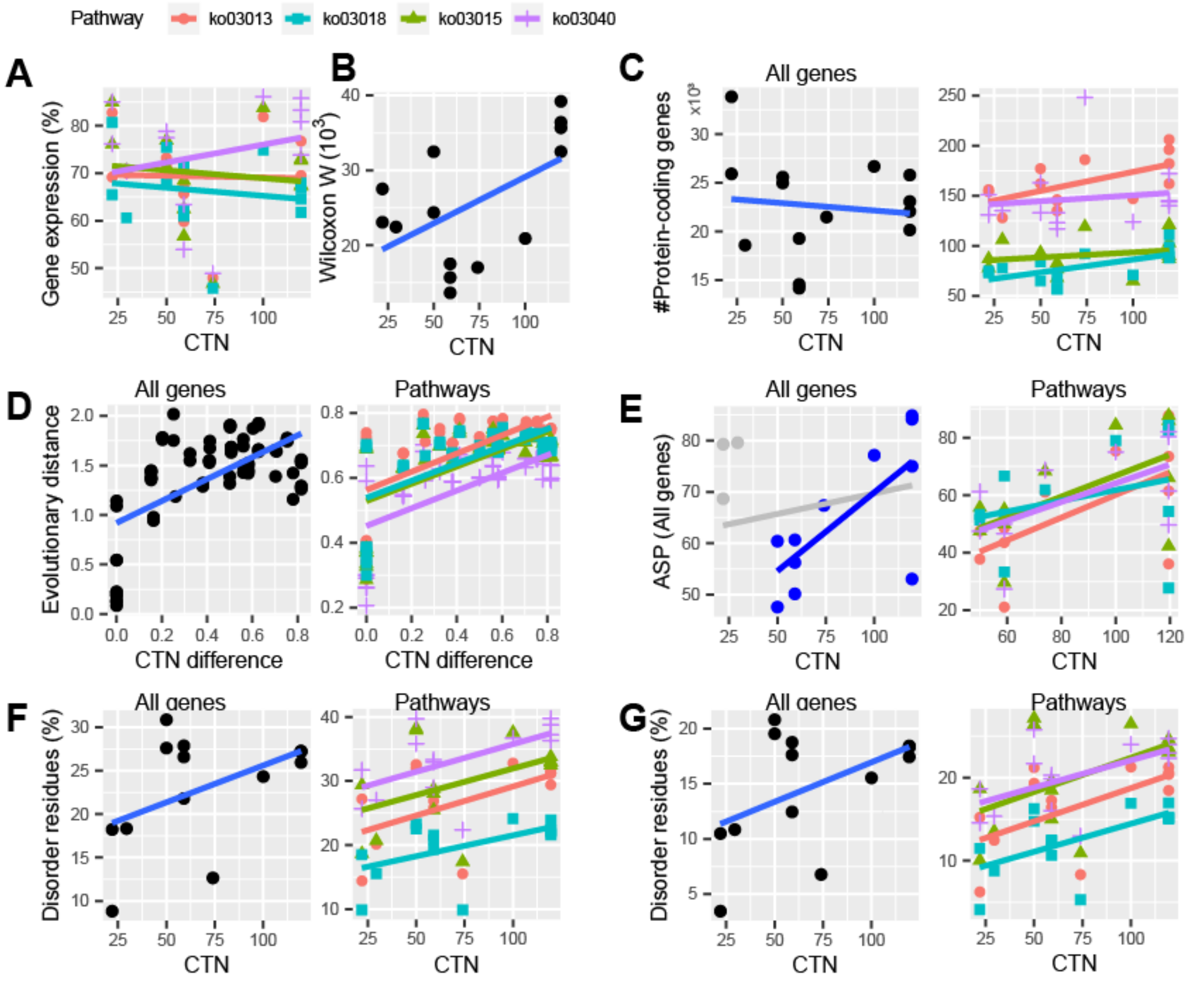

Supplementary Fig. S7. Relationships between organism complexity and the six genomic features of splicing factors in the SecondSpeciesSet. The analysis details are the same as those described in the figure legend of Fig 5. The four ASP/L-related pathways investigated were RNA transport (ko03013), mRNA surveillance pathway (ko03015), RNA degradation (ko03018), and spliceosome (ko03040). The gene expression levels were calculated using LeafCutter (A), and, for each species, the Wilcoxon W-statistics was calculated between ko03040 and the combination of the other three pathways (B), which yielded Spearman's ρ = 0.39, P = 0.17. (C) Scatter plots of the genome-wide protein-coding genes (left) and those along the four pathways (right) vs. the cell type number (CTN). The gene numbers in the four pathways were positively correlated with organism complexity using the genome-wide protein-coding genes as the background (Chi-square test: ko03040, ko03013, ko03018, and ko03015, P = 5.8e-26, 6.9e-15, 6.3e-07, and 7.7e-12, respectively). (D) Scatter plots of the evolutionary distance and organism complexity differences across the 14 species based on the species phylogenetic tree and the protein alignment of the KEGG orthologs along the four pathways

(right) vs. the CTN difference. The CTN difference was calculated as diff(a,b)/max(a,b) and ranged between 0 and 1. The evolutionary distances were positively correlated with the organism complexity differences (left: Spearman's ρ = 0.4, *P* = 6.5e-05; right: for ko03040, ko03013, ko03015, and ko03018, ρ = 0.49, 0.43, 0.44, and 0.44, respectively, and *P* = 1.4e-04, 1.2e-03, 7.8e-04, and 8.5e-04, respectively). (E) Positive correlations between ASP and CTN using the LeafCutter tool for all genes (left) and the genes along the four pathways (right) (left: Spearman's ρ = 0.66, *P* = 0.03; right: for ko03013, ko03015, ko03018, and ko03040, Spearman's ρ = 0.54, 0.5, 0.42, and 0.61, respectively, and *P* = 0.09, 0.11, 0.2, and 0.05, respectively). (F) Positive correlations between the fraction of disordered residues and CTN for all genes (left: Spearman's ρ = 0.31, *P* = 0.28) and genes along the four pathways (right: for ko03013, ko03015, ko03018 and ko03040, Spearman's ρ = 0.38, 0.3, 0.52, and 0.58, respectively, and *P* = 0.19, 0.31, 0.06, and 0.03, respectively). (G) Scatter plots of the fraction of binding residues vs. CTN for all genes (left: Spearman's ρ = 0.3, *P* = 0.3) and genes along the four pathways (right: for ko03013, ko03015, ko03018 and ko03040, Spearman's ρ = 0.55, 0.38, 0.62, and 0.52, respectively, and *P* = 0.04, 0.19, 0.02, and 0.06, respectively).

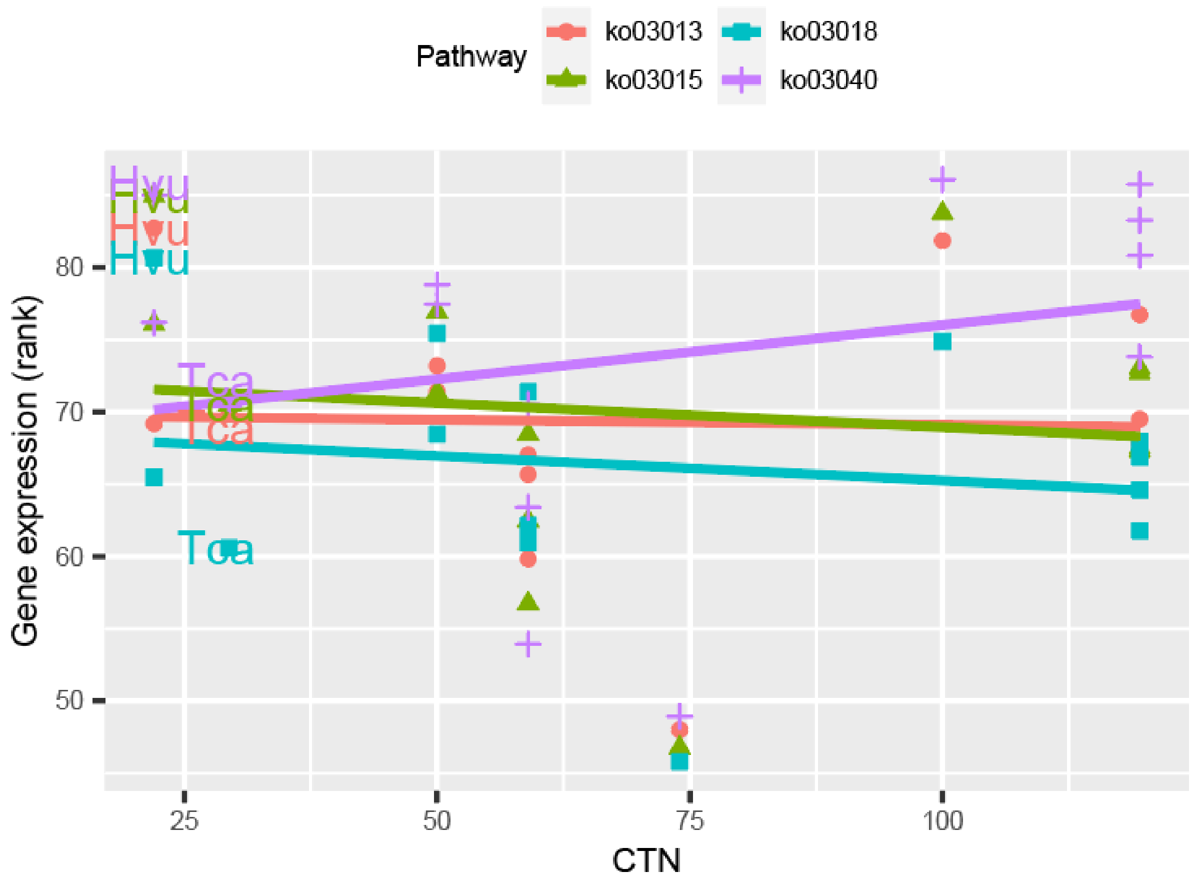

Supplementary Fig. S7A. This version of Supplementary Fig. S7 panel (A) includes Hvu and Tca. CTN, cell type number.
