# Supplementary material for: Alternative splicing level related to intron size and organism complexity
Source: BMC Genomics. 2021 Nov 25;22:853. doi: 10.1186/s12864-021-08172-2 (PMC8614042; doi:10.1186/s12864-021-08172-2)
Supplement: Supplementary file 16 — Additional file 16: Figure S8. Correlation among genomic features of ASP/L-related genes in the SecondSpeciesSet. (A) Intersection of significant KOs among six genomic features. Spearman correlation and PGLS regression were calculated between these genomic features and organism complexity for each KO in the SecondSpeciesSet. Significant KOs were defined as Spearman’s ρ > 0.4 and PGLS P < 0.05. (B) Principal component analysis of these six genomic features based on the Spearman’s ρ in 112 KOs. The points represent KOs and the shapes represent the four pathways. [file 12864_2021_8172_MOESM16_ESM.pdf]

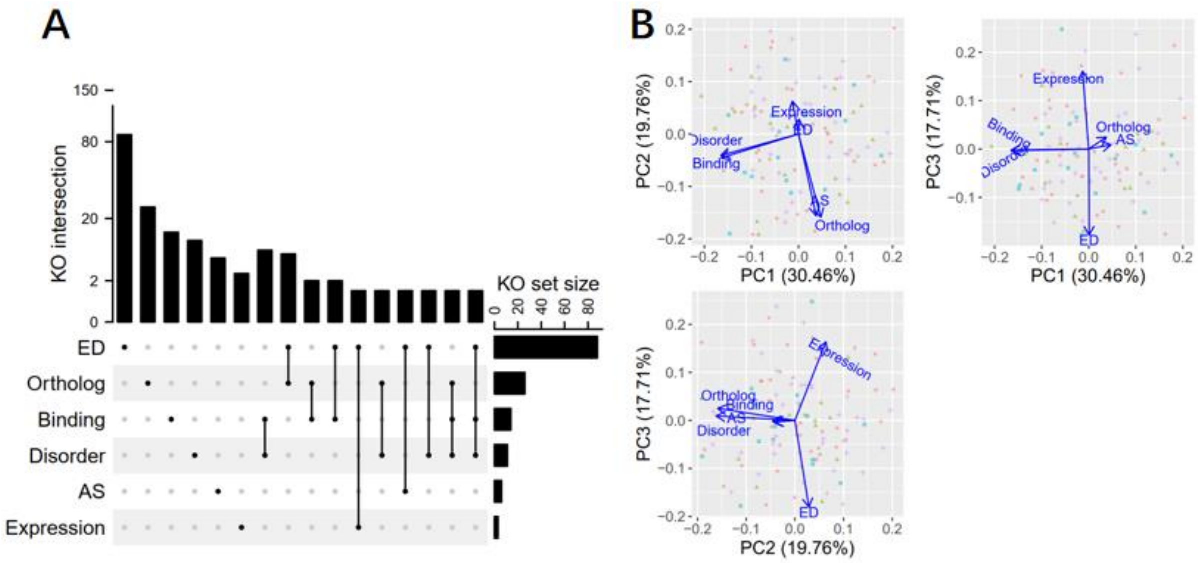

Supplementary Fig. S8. Correlation among genomic features of ASP/L-related genes in the SecondSpeciesSet. (A) Intersection of significant KOs among six genomic features. Spearman correlation and PGLS regression were calculated between these genomic features and organism complexity for each KO in the SecondSpeciesSet. Significant KOs were defined as Spearman's ρ > 0.4 and PGLS P < 0.05. (B) Principal component analysis of these six genomic features based on the Spearman's ρ in 112 KOs. The points represent KOs and the shapes represent the four pathways.
