# Supplementary material for: Alternative splicing level related to intron size and organism complexity
Source: BMC Genomics. 2021 Nov 25;22:853. doi: 10.1186/s12864-021-08172-2 (PMC8614042; doi:10.1186/s12864-021-08172-2)
Supplement: Supplementary file 8 — Additional file 8: Figure S2. ASP/L displayed a positive correlation with organism complexity when using the D5 and D6 datasets. The boxplot indicates the P value with -log10 transformation for each column. PASP, predicted alternative splicing prevalence; ASP, actual alternative splicing prevalence; PASL, predicted alternative splicing level; ASL, actual alternative splicing level; GeneNum, gene number; SplicLvl, splicing level; CorMethod, correlation calculation method. [file 12864_2021_8172_MOESM8_ESM.pdf]

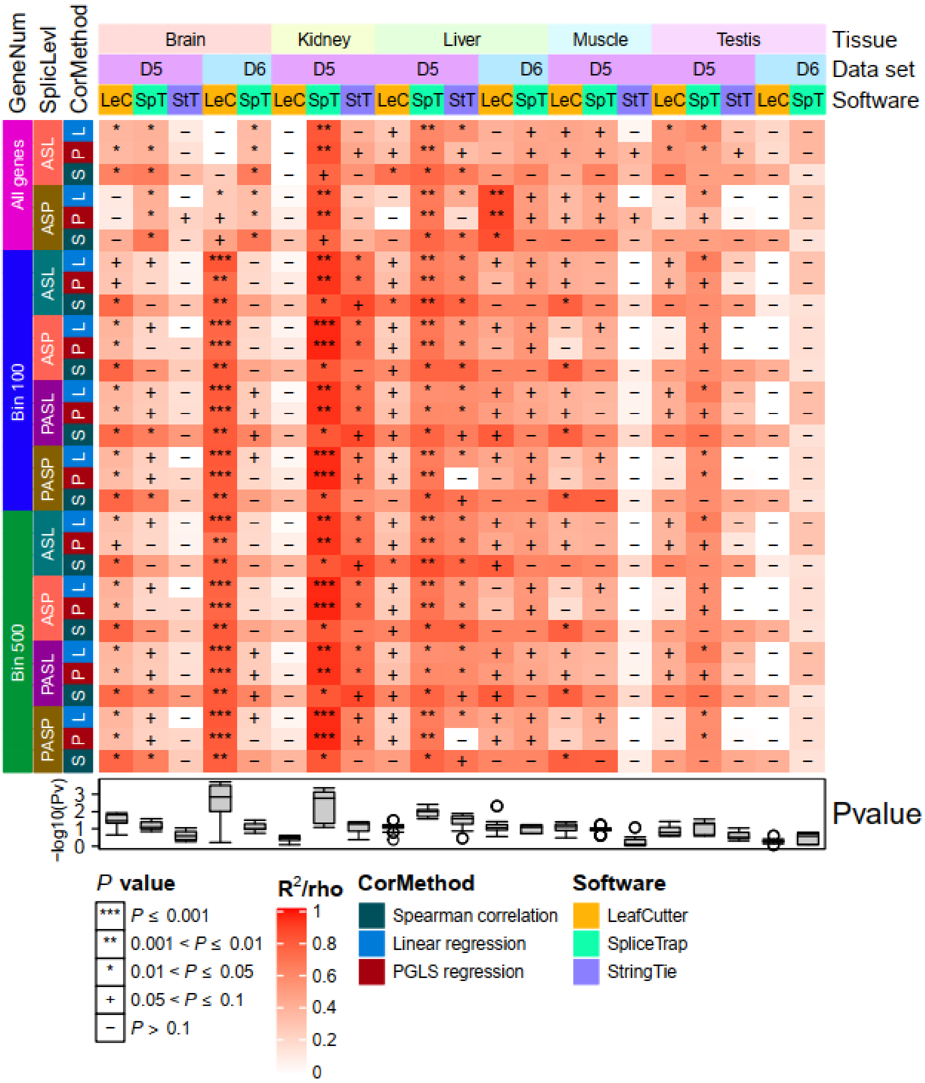

Supplementary Fig. S2. ASP/L displayed a positive correlation with organism complexity when using the D5 and D6 datasets. The boxplot indicates the *P* value with –log10 transformation for each column. PASP, predicted alternative splicing prevalence; ASP, actual alternative splicing prevalence; PASL, predicted alternative splicing level; ASL, actual alternative splicing level; GeneNum, gene number; SplicLvl, splicing level; CorMethod, correlation calculation method.
